# In Situ Halide Vacancy Tuning of Low‐Dimensional Lead Perovskites to Realize Multiple Adjustable Luminescence Performance

**DOI:** 10.1002/advs.202412459

**Published:** 2025-03-17

**Authors:** Chen Sun, Chang‐Qing Jing, Dong‐Yang Li, Meng‐Han Dong, Ming‐Xue An, Zhong‐Hui Zhang, Cheng‐Yang Yue, Honghan Fei, Xiao‐Wu Lei

**Affiliations:** ^1^ Research Institute of Optoelectronic Functional Materials, School of Chemistry Chemical Engineering and Materials Jining University Qufu Shandong 273155 China; ^2^ Shanghai Key Laboratory of Chemical Assessment and Sustainability School of Chemical Science and Engineering Tongji University 1239 Siping Rd. Shanghai 200092 China; ^3^ Jiangsu Key Laboratory for Science and Applications of Molecular Ferroelectrics and School of Chemistry and Chemical Engineering Southeast University Nanjing 211189 China

**Keywords:** 0D organic‐inorganic halide perovskites, adjustable emission, defect passivation, halogen vacancies

## Abstract

Surface defects play a crucial role in the photophysical properties and optoelectronic applications of perovskite materials. Although luminescent efficiency is improved through post‐synthetic defect passivation, comprehensive optimization of photoluminescent performance via defect chemistry remains a significant challenge. Herein, a successful defect engineering strategy is demonstrated toward 0D perovskite of [DADPA]PbBr_5_ (DADPA = diaminodipropylamine) single crystal to achieve multiple adjustable luminescent properties. Through fine‐tuning the crystallization environment to diminish Br vacancy (*V*_Br_), [DADPA]PbBr_5_ displays gradually evolutionary luminescence range from broadband blue‐white to narrow green light emissions, with continuously adjustable dominant wavelengths (445–535 nm) and linewidths (134–27 nm). Meanwhile, the quantum yields increase significantly from 3.7% to 80.8%, and lifetime extends from 5.4 to 57.7 ns. This is the pioneering discovery in perovskite chemistry for simultaneous modification of multi‐dimensional luminescent performances. Combined spectroscopic investigations and first‐principles calculations indicate that the reducing *V*_Br_ significantly narrows the bandgap and inhibits nonradiative recombination, which attenuates interband trap‐state‐associated broadband emission and facilitates the formation of bound exciton for enhanced emission efficiency. More remarkably, this universal strategy can be extended to other perovskite systems with similar luminescent adjustability, paving the way for applications of diverse perovskites with improved optoelectronic performance.

## Graphical abstract

A novel surface defect engineering strategy toward low‐dimensional halide perovskite is proposed to achieve multiple optimized luminescent properties including continuously adjustable bandgap and emission color in a wide range, varied emission linewidth, increased PLQY, and extended lifetime. The in‐depth investigations further provide a significant understanding of integrated defect‐band structure‐property relationship of perovskite materials. This achievement first highlights a proof‐of‐concept luminescent modification strategy expanding the scientific horizon of luminescent perovskite materials.

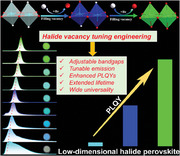

## 1 Introduction

Crystalline semiconducting materials inevitably contain various shallow or internal defects with discontinuously periodic atomic arrangements including vacancy, antisite, dislocation and grain boundary, etc.^[^ ^1^ ^]^ These structural defects readily give rise to local electronic band structural distortion and interband energy states relative to the long‐range ordered bulk crystal and play crucial influence on the electrical and optical properties of semiconducting materials.^[^ ^2^ ^]^ For example, the surface atomic vacancy can efficiently accelerate the separation of photo‐induced electron‐hole pairs and dramatically improve the catalytic activity of semiconducting photocatalysis.^[^ ^3^ ^]^ Surface defect engineering has also realized astonishing breakthroughs in photovoltaic efficiency of solar cell technology in Si, GaAs, Cu(In,Ga)Se_2_, CsPbX_3_, etc.^[^ ^4^ ^]^ Especially, the surface defect influences the exciton population and creates disorder‐induced localization, providing new opportunity to tailor the photoluminescent (PL) properties of materials. Conventional examples include nitrogen‐vacancy defect‐induced fluorescence in diamond,^[^ ^5^ ^]^ green/blue emission from indium dislocation of InGaN,^[^ ^6^ ^]^ oxygen vacancy associated broadband emission of ZnO,^[^ ^7^ ^]^ tunable single‐photon emission in hexagonal boron nitride (h‐BN) and carbon nanotube from various atomic defects,^[^ ^8^ ^]^ quantum optical properties of transition metal dichalcogenides due to chalcogen vacancy, etc.^[^ ^9^ ^]^ In this context, surface defect enriches the functionalities of semiconducting materials, and defect engineering chemistry opens a new opportunity for advanced optoelectronic optimization of semiconductors at the ultimate scale.

In recent years, 3D lead perovskite nanocrystals (PNCs) have emerged as new‐generation semiconductors with multiple merits of facile assembly process, high defect tolerance, adjustable band gap, long carrier diffusion length and lifetime, etc.^[^ ^10^ ^]^ These distinctive physical properties endow PNCs with advanced applications in various photoelectric devices of solar cells, light‐emitting diode (LED), photodetectors, lasers, etc.^[^ ^11^ ^]^ Especially, the all‐inorganic CsPbX_3_ (X = Cl, Br, and I) nanocrystals display outstanding PL performance with combined advantages of adjustable emission wavelength in the whole visible spectral range, high photoluminescence quantum yield (PLQY ≈ 90%), narrow emission bandwidth (12–25 nm) and wide color gamut (≈150% NTSC, NTSC = National Television Standards Committee), etc.^[^ ^12^ ^]^ Although 3D perovskites show higher tolerance to the surface defects than conventional semiconductor NCs (i.e., InP and CdSe),^[^ ^13^ ^]^ the solution process synthesized PNCs inevitably contain a certain amount of defects during the rapid crystallization process including halide dangling bonds or interstitial, lead vacancy, grain boundary and so on, because of highly dynamic ionic binding nature. Due to the exciton quenching of these defects, 3D PNCs still suffer from defects‐mediated nonradiative losses and spectral instabilities, which severely restrict their practical applications in solid‐state LEDs.^[^ ^14^ ^]^ To reduce the defect density and improve the PLQY and stability, the most effective method is utilizing the proper molecular passivators with different anchoring groups to polish the surface and eliminate the defect points. Until now, a wide range of organic and inorganic salts have been adopted to passivate the surface through coordinating bond to exposed lead ions to enhance hydrogen bonding with halides and stabilize PNCs on the basis of Lewis acid‐base theory, such as long‐chain alkylamines and alkyl acids, thiocyanate salts, metal halide salts, fluoride, acetate, carbonyl‐, phosphine‐ and sulfur‐based ligands, etc.^[^ ^15^ ^]^ Especially, some traditional ammonium ligands, such as phenylethyl ammonium, bond to exposed lead ions through strengthening weak hydrogen bonding with halides, providing limited binding force for the anchoring lattice.^[^ ^16^ ^]^ Significantly, this post‐synthetic surface passivation strategy not only enhances the emission efficiency and boosts the PL lifetime but also enhances the photophysical stability of 3D PNCs.^[^ ^17^ ^]^ Unfortunately, the emission wavelength of 3D PNCs remains to locate near the band edge and can't change with defect contents due to the quenching of new sub‐band trap states associated recombination transitions. The detailed defect tuning mechanism is still unclear. Therefore, it is significant but challenging to realize all‐around modification of PL performance through synchronously fine‐tuning the energy levels of surface defects in perovskite materials on the basis of understanding the defect‐property relationship.

Recently, significant achievements of 3D PNCs simultaneously inspire the rapid development of low‐dimensional organic‐inorganic halide perovskites (OIHPs), which can be considered as the derivatives of 3D perovskite.^[^ ^18^ ^]^ These low‐dimensional OIHPs attracted extensive academic attention as one of the most promising luminescent materials due to multiple merits including non‐toxicity, diversified structural architectures and chemical components, high exciton binding energy and emission efficiency, and so on.^[^ ^19^ ^]^ Comparing with 3D PNCs, the single‐crystalline low‐dimensional OIHPs are inclined to display broadband light emissions from self‐trapped excitons (STEs) with more tunable PL properties.^[^ ^20^ ^]^ Indeed, the STEs can be considered as “excited‐state defects”, that is, the electrons and phonons are strongly coupled to form transient elastic lattice distortions, which rapidly trap the excited‐state electrons to release the energy as recombination transition.^[^ ^21^ ^]^ Apart from the intrinsic excited‐state defects associated STE states, the photoinduced electrons are also easily trapped by crystal defects as broadband light emissions resembling those of typical semiconducting materials of ZnO and h‐BN.^[^ ^7^, ^8^ ^]^ For example, 0D (C_9_NH_20_)_6_Pb_3_Br_12_ displays broadband green light emission arising from the sub‐bandgap transitions of permanent Br vacancy (*V*_Br_) rather than STEs of excited‐state structural reorganization.^[^ ^22^ ^]^ 1D C_4_N_2_H_14_PbCl_4_ exhibits dual broadband bluish‐green and yellow light emissions from STEs and vacancy‐bound excitons, respectively.^[^ ^23^ ^]^ More remarkably, by rationally increasing the density of Sn^2+^ vacancy to enhance the exciton localization, the PLQY of 2D(2D) (OA)_2_SnI_4_ (OA = octylammonium) can be optimized from 7.0% to 63.6%.^[^ ^24^ ^]^

**Scheme 1:**
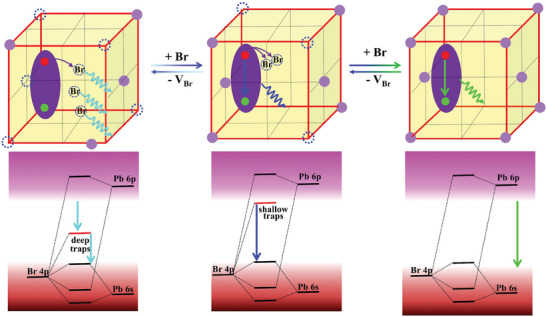
Schematic illustration of surface defect engineering on exciton diffusion processes, electronic in‐gap states, and luminescence transformation in lead halide perovskites. The dashed circles represent *V*_Br_.

These achievements imply that the surface defects play crucial role in the excitonic distribution characteristics of low‐dimensional OIHPs, which provides additional modification channels to regulate their PL properties. However, this phenomenon is nearly ignored over a long period of time due to ambiguous regulating mechanisms and difficult tuning methods, and rare effective synthetic technique is capable to activate the surface defects as independent emitting centers, let alone the adjustable luminescent properties. Herein, for the first time, we performed a facile *V*_Br_ tuning strategy on 0D [DADPA]PbBr_5_ (DADPA = diaminodipropylamine) to dynamically control the electronic band structure and PL properties (**Scheme** **1**). Specifically, through controlling the passivation effects of synthetic solvent during crystallization to decrease the content of *V*_Br_ and further gradually suppress the defect trap states and facilitate the intrinsic bound excitions of perovskite lattice, the defect‐associated broadband emission of [DADPA]PbBr_5_ turns into narrow band‐edge green emission step by step with varied wavelength from 445 to 535 nm and increased PLQY from 3.7% to 80.8%. Based on the in‐depth investigation of underlying mechanism, this proof‐of‐concept strategy is successfully extended to other low‐dimensional OIHPs opening a novel window for designing more desirable luminescent perovskite materials. This work represents the first surface engineering strategy to achieve multiple adjustable luminescent performances in perovskite chemistry.

## 2 Results and Discussion

**Figure 1:**
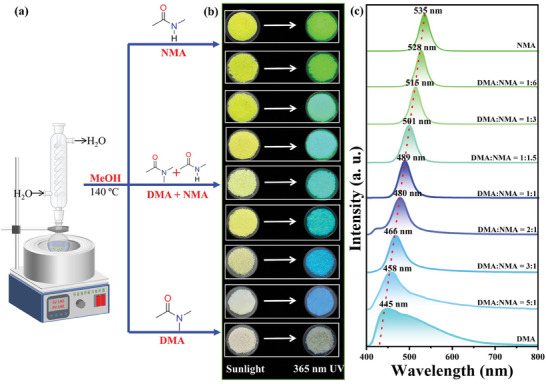
a) Solvothermal preparation process for serial [DADPA]PbBr_5_ single crystals; b) corresponding photo images under sunlight and 365 nm UV light; c) evolutional PL emission spectra from white to green light emission.

Previous works have demonstrated that halogen vacancy plays important role in excitonic distribution and electronic band of single‐crystalline OIHPs, which provides the feasibility to regulate their luminescent properties.^[^ ^25^ ^]^ To validate this defect engineering strategy, we chose a new 0D hybrid lead perovskite of [DADPA]PbBr_5_ as initial structural model with controllable content of *V*_Br_. For this purpose, we synthesized a series of 0D [DADPA]PbBr_5_ single crystals (SCs) containing different content of apparent *V*_Br_ by a modified solvothermal reaction with the same precursor materials but different feeding ratios of N,N‐dimethylacetamide (DMA) and N‐Methylacetamide (NMA) from 1: 0 to 0: 1 (**Figure** **1**a). Through fine‐tuning the crystallization environment, all the obtained [DADPA]PbBr_5_ SCs crystallize in the same crystal lattice with identical powder X‐ray diffraction (PXRD) patterns, but the appearances change from colorless, yellow to yellow‐green colors indicating different optical absorption characteristics (Figure 1b; Figure S1, Supporting Information). More remarkably, the [DADPA]PbBr_5_ SCs display continuously adjustable luminescence in wide range from weak white to strong blue and green light emission under the irradiation of 365 nm UV light, which can be easily distinguished by naked eye (Figure 1b). Detailed PL emission spectra indicate that the dominant emission wavelength significantly shifts from 445 to 535 nm accompanied by gradually narrowed full width at half maximum (FWHM) from 134 to 27 nm and enhanced PLQY from 3.7% to 80.8% (Figure 1c). Therefore, diversified PL properties including adjustable emission wavelength, FWHM, and efficiency are realized simultaneously in the same [DADPA]PbBr_5_ structural model. To understand the adjustable luminescent performance and underlying mechanism of this unique halide family, systematical chemical composition and spectroscopic characterizations as well as theoretical calculations were performed.

**Figure 2:**
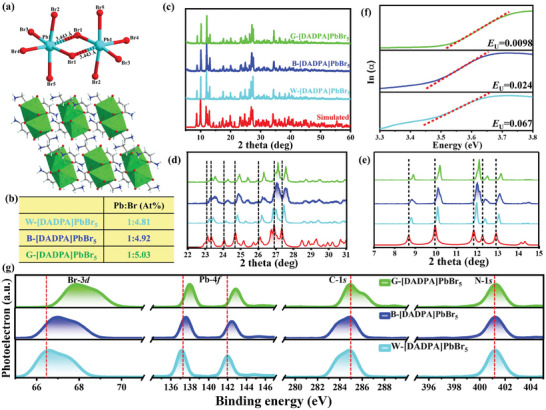
a) The crystal structures of [Pb_2_Br_10_]^6−^ dimers (up) and packing crystal structure (down) of [DADPA]PbBr_5_; b) average ratio of Pb and Br based on the ICP results (atom %); c) powder X‐ray diffraction patterns of W‐, B‐, and G‐[DADPA]PbBr_5_; d,e) detailed comparison of powder X‐ray diffraction patterns between 22–31° and 7–15° for W‐, B‐, and G‐[DADPA]PbBr_5_; f) logarithmic absorption coefficient extracted from optical absorption spectra as a function of photon energy, and corresponding Urbach energies for W‐, B‐, and G‐[DADPA]PbBr_5_; g) comparison of the HRXPS peaks for Br‐3*d*, Pb‐4*f*, C‐1*s*, N‐1*s* of W‐, B‐, and G‐ [DADPA]PbBr_5_.

For simplicity, the white, blue, and green emissive [DADPA]PbBr_5_ SCs were selected as representatives and defined as W‐[DADPA]PbBr_5_, B‐[DADPA]PbBr_5_ and G‐[DADPA]PbBr_5_, respectively. Single crystal X‐ray diffractions demonstrate that the selected three [DADPA]PbBr_5_ SCs crystallize in the same monoclinic crystallographic space group of *P*2_1_ */c* with the same crystal structures (Table S4, Supporting Information). The structure is composed of bulk [DADPA]^3+^ and discrete [Pb_2_Br_10_]^6−^ dimer from edge‐shared [PbBr_6_] octahedron (**Figure** **2**a). The [Pb_2_Br_10_]^6−^ dimers are surrounded and separated by [DADPA]^3+^ to form long‐range ordered 0D hybrid structure through abundant weak hydrogen bonds and electrostatic interactions. This structure can be considered as semiconducting inorganic units periodically embedded in insulating organic matrix as individual optical centers. All the peaks in Fourier‐transform Infrared (FT‐IR) and Raman spectra are identical indicating the same band interactions for three [DADPA]PbBr_5_ SCs (Figures S2 and S3, Supporting Information). Inductively coupled plasma‐mass spectrometry (ICP‐MS), energy dispersive X‐ray spectroscopy (EDX), and elemental mapping analyses were performed to confirm the uniform distribution of all elements including C, N, Pb, and Br on the surface of single crystals (Figures S4–S6, Supporting Information). However, the actual Br: Pb mole ratio of ≈4.81: 1 for W‐[DADPA]PbBr_5_ is far below than the stoichiometric ratio (5.0: 1) in the ideal molecular formula indicating the high *V*_Br_ density in the crystal lattice. Comparing with W‐[DADPA]PbBr_5_, B‐ and G‐[DADPA]PbBr_5_ have higher Br:Pb mole ratios of ≈4.92: 1 and ≈5.03: 1 indicating significantly decreased Br vacancy, and the content of *V*_Br_ follow the order of W‐[DADPA]PbBr_5_ > B‐[DADPA]PbBr_5_ > G‐[DADPA]PbBr_5_ (Figure 2b). The different content of *V*_Br_ is further confirmed by subsequent characterizations. All the PXRD patterns of three [DADPA]PbBr_5_ SCs match well with the simulated data without any additional peaks indicating the same crystal structures (Figure 2c). Careful inspection shows a slight XRD peak shifting to lower angle indicating the crystal lattice expansion from W‐ to G‐[DADPA]PbBr_5_ SCs owing to the slightly increased content of Br element (Figure 2d,e). To further clarify this phenomenon, Urbach energy (*E*_U_) was adopted to evaluate the degree of bromine vacancy by plotting the absorption coefficient as a function of photon energy. In general, impurity or defect of semiconducting materials results in crystal disorder, which is related to the UV‐vis energy absorption edge and Urbach tail.^[^ ^26^ ^]^ The less crystal impurity or defect means the short absorption tail and small *E*_U_. Now, *E*_U_ is calculated by fitting the exponential part of Urbach tail according to the following Equation (1):

(1) $$\alpha \left(E\right)=\alpha_{0}exp\left[\sigma \left(T\right)\frac{E-E_{0}}{k_{B}T}\right]$$

where *k*_B_ is the Boltzmann constant, *T* is the absolute temperature, and *E*_U_ is defined as *E*_U_ = *k*_B_ *T*/*σ*(*T*).^[^ ^27^ ^]^ As shown in Figure 2f, the calculated *E*_U_ values of 0.067, 0.024, and 0.0098 eV follow the order of W‐[DADPA]PbBr_5_ > B‐[DADPA]PbBr_5_ > G‐[DADPA]PbBr_5_, which indicates the decreased *V*_Br_ content from W‐ to B‐ and G‐[DADPA]PbBr_5_. Moreover, the X‐ray photoelectron spectroscopy (XPS) measurements show that no peak shift is observed for C‐1*s* and N‐1*s* in all phases indicating the negligible effect of the crystallization environment on organic cations (Figure 2g). However, the peaks of Pb‐4*f*_5/2_ and Pb‐4*f*_7/2_ as well as Br‐3*d*_3/2_ and Br‐3*d*_5/2_ gradually shift to higher binding energies from W‐ to B‐ and G‐[DADPA]PbBr_5_ SCs owing to stronger Pb‐Br interaction, which attest the gradual elimination of *V*_Br_ in [DADPA]PbBr_5_. Together these investigations validate the gradually decreased *V*_Br_ density from W‐ to B‐ and G‐[DADPA]PbBr_5_ SCs, which demonstrate the successful controlling of *V*_Br_ density through rationally fine‐tuning the crystallization condition.

**Figure 3:**
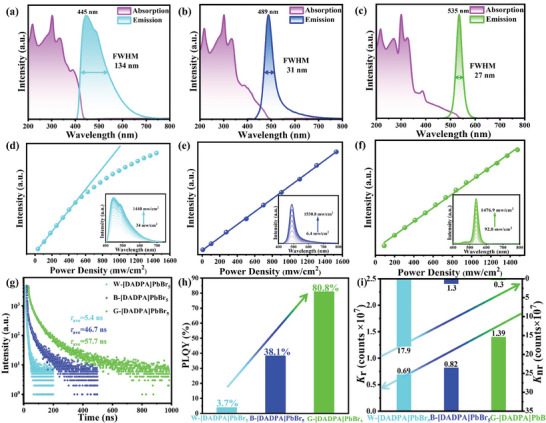
Systematical characterizations of PL properties for W‐, B‐ and G‐[DADPA]PbBr_5_; a–c) absorption and emission spectra of W‐, B‐ and G‐[DADPA]PbBr_5_ respectively; d–f) power density dependent PL emission intensity of W‐, B‐ and G‐[DADPA]PbBr_5_ respectively; g) time‐resolved PL spectroscopy of W‐, B‐ and G‐[DADPA]PbBr_5_; h) the absolute PLQYs of W‐, B‐ and G‐[DADPA]PbBr_5_; i) the radiative and nonradiative recombination rate (*K*_r_ and *K*_nr_) for W‐, B‐ and G‐[DADPA]PbBr_5_.

The optical absorption and PL spectra were measured at room temperature to reveal the effect of *V*_Br_ on the photophysical properties of serial [DADPA]PbBr_5_ phases. As shown in **Figure** **3**a–c, the UV‐vis absorption spectra give similar intensive absorption bands below 350 nm arising from the interband electronic absorption with weak broad absorption from 350 to 425 nm, 500 and 550 nm for W‐, B‐ and G‐[DADPA]PbBr_5_, respectively. The slight red shift of absorption onsets from W‐ to G‐[DADPA]PbBr_5_ could be corresponding to the enhanced band edge state density resulting from decreased *V*_Br_. To more accurately evaluate the defect density, we plotted the absorption spectra toward photon energy in direct‐bandgap mode according to the Kubelka‐Munk method (Figure S7, Supporting Information). Obviously, G‐[DADPA]PbBr_5_ has the narrowest Urbach tail, while W‐[DADPA]PbBr_5_ gives more broader absorption tail at low energies corresponding to more crystal defects.^[^ ^27^ ^]^ Upon the excitation of 372 nm UV light, W‐[DADPA]PbBr_5_ displays broadband light emission with one dominant peak at 445 nm and one weak shoulder peak at ≈488 nm as well as FWHM of 134 nm (Figure 3a). By contrast, B‐ and G‐[DADPA]PbBr_5_ exhibit narrow blue and green light emissions centered at ≈489 and 535 nm with FWHM of 31 and 27 nm, respectively (Figure 3a,b). The varied excitation wavelength‐dependent PL spectra indicate the changeless emission spectral profiles in the whole UV light range, and 3D consecutive PL maps also give only one emitting center for each phase indicating the single radiative recombination pathway (Figures S8–S11, Supporting Information). To comprehend the individual photophysical mechanism, we then performed in‐depth spectroscopy characterizations for three compounds. In conventional semiconducting materials such as ZnO and CdSe, the light emission can be attributed to the mid‐bandgap states arising from the deep traps at surface sites, and the emission intensity is strongly dependent on the particle size.^[^ ^28^, ^29^ ^]^ Hence, we first compare the PL emission spectra of three samples with different particle sizes (<5, 50, and 100 µm) at equivalent loading amounts (Figure S12, Supporting Information). As shown in Figure S13 (Supporting Information), the PL emission intensities of B‐ and G‐[DADPA]PbBr_5_ are nearly independent on the particle sizes, while the emission intensity of W‐[DADPA]PbBr_5_ decline gradually with the decreasing of particle size possibly due to the fact that smaller particle size tends to possess larger surface area and higher concentration of *V*_Br_, which seriously attenuate the luminescence efficiency.^[^ ^30^ ^]^ Furthermore, the excitation power density‐dependent PL emission spectra were performed. The emission intensity of W‐[DADPA]PbBr_5_ features a saturation behavior indicating that the finite *V*_Br_ is filled by photo‐excited electrons with excitation light intensity increasing (Figure 3d). Differently, the PL emission intensities of B‐ and G‐[DADPA]PbBr_5_ exhibit linear dependence on the excitation power density excluding the possibilities of permanent defect‐induced emission (Figure 3e,f). Together these results successfully demonstrate the participation of bromine vacancy defect in broadband light emission of W‐[DADPA]PbBr_5_ but negligible effect on B‐ and G‐[DADPA]PbBr_5_. Considering the dominant emission peaks of B‐ and G‐[DADPA]PbBr_5_ located near the absorption onsets, the narrow blue and green emissions can be ascribed to the band‐edge recombination of free excitons (FEs). By contrast, the broadband white light emission of W‐[DADPA]PbBr_5_ is mainly contributed by the *V*_Br_ defect associated with sub‐bandgap trap states.

Subsequently, time‐resolved PL spectroscopy was further used to examine the carrier recombination dynamics of these emissions. For W‐[DADPA]PbBr_5_, the PL decay curves give an average lifetime of 5.4 and 4.1 ns monitoring at 445 and 488 nm, respectively, based on double‐exponential function (Figure 3g; Figure S14, Supporting Information). Comparing with W‐[DADPA]PbBr_5_, the lifetime of B‐ and G‐[DADPA]PbBr_5_ extend to 46.7 and 57.7 ns indicating the more distributions of excited state electrons and suppressed nonradiative process after the decreasing of *V*_Br_ (Figure 3g). In addition to the red‐shift of emission wavelength, broadening of FWHM, and prolongation of lifetime, the decreased *V*_Br_ also significantly improves the PL emission efficiency, which is consistent with the typical defect‐passivated 3D PNCs. As shown in Figure 3h, the absolute PLQY increases from 3.7%, 38.1% to 80.8% for W‐, B‐ and G‐[DADPA]PbBr_5_ with gradual decreasing content of *V*_Br_, which implies that the defect‐assisted non‐radiative process is effectively suppressed (Figure S15, Supporting Information). Subsequently, the radiative and nonradiative recombination rates (*K*_r_ and *K*_nr_) were evaluated based on PLQY (*φ*) and lifetime (*τ*) according to Equations (2) and (3):

(2) $$K_{r}=\frac{\varphi }{\tau }$$

(3) $$K_{\mathrm{nr}}=\frac{1-\varphi }{\tau }$$

As summarized in **Table** **1**, the *K*_r_ gradually increases from 0.69 × 10^7^ to 1.39 × 10^7^ s^−1^ and *K*_nr_ simultaneously declines from 17.9 × 10^7^ to 0.3 × 10^7^ s^−1^ along with the decreased *V*_Br_ from W‐, B‐ to G‐[DADPA]PbBr_5_ (Figure 3i). The increased *K*_r_ and decreased *K*_nr_ suggest that the enhanced PLQY is primarily due to the enhanced radiative decay process and suppressed nonradiative recombination arising from the reduced *V*_Br_. Despite the PLQY of typical 3D PNCs can be improved by decreasing the surface defect density in previous work, no successful synthetic technology has been realized to tailor the emission spectral range yet. To the best of our knowledge, this is the first report about the synchronously adjustable emission wavelength, linewidth, lifetime, and emission efficiency through engineering halide vacancy in perovskite materials, which provides a novel structural design strategy to optimize PL performance.

**Table 1:** The summary of photophysical properties for W‐, B‐ to G‐[DADPA]PbBr_5_.

	*λ*_em_ [nm]	FWHM [nm]	PLQY [%]	Lifetime [ns]	*K*_r_ (×10^7^) [s^−1]^	*K_n_*_r_ (×10^7^) [s^−1^]
W‐[DADPA]PbBr_5_	445	134	3.7	5.4	0.69	17.9
B‐[DADPA]PbBr_5_	489	31	38.1	46.7	0.82	1.3
G‐[DADPA]PbBr_5_	535	27	80.8	57.7	1.39	0.3

**Figure 4:**
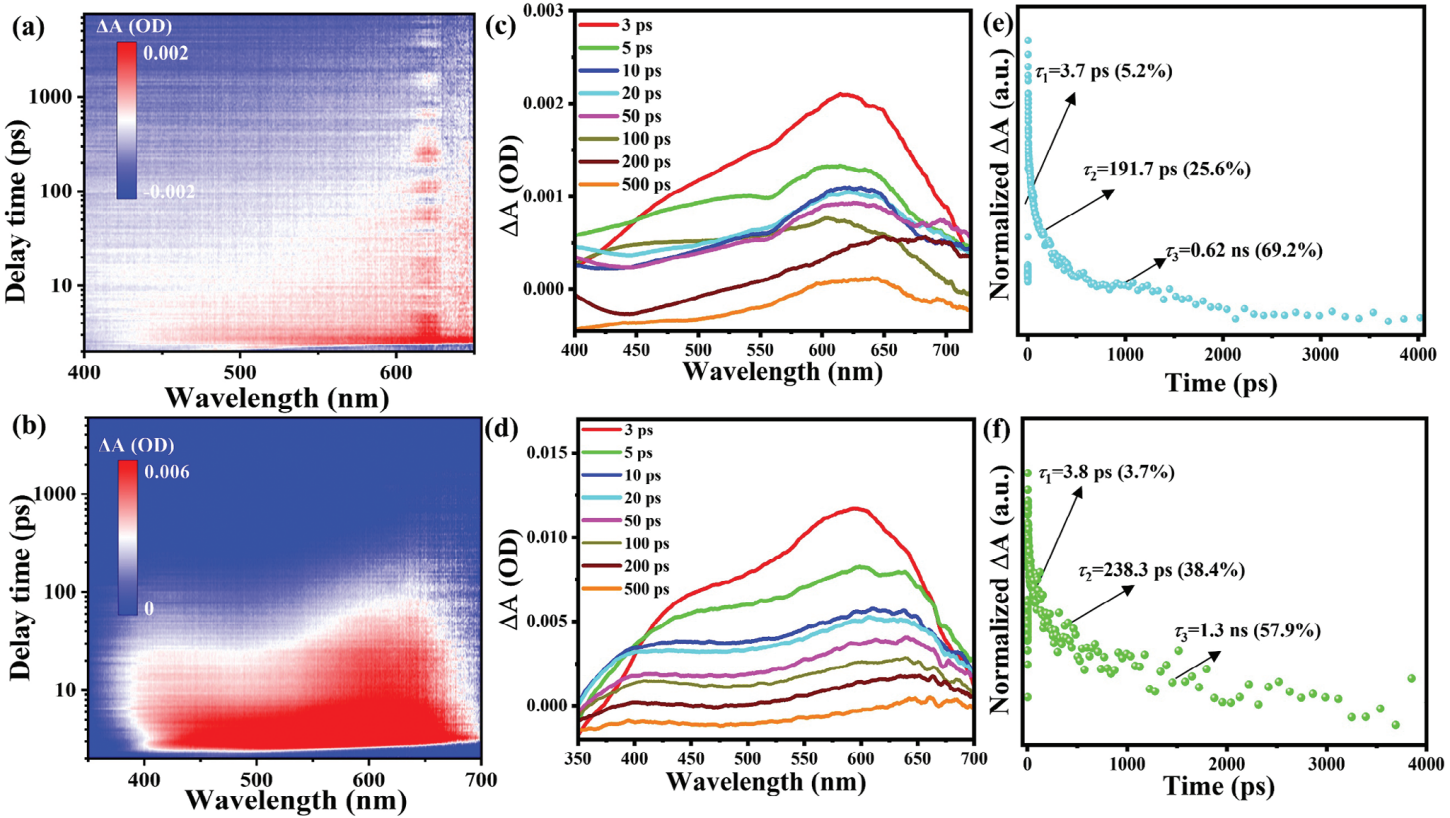
Fs‐TA spectra of W‐ (up) and G‐[DADPA]PbBr_5_ (down): a and d) Pseudo contour plot as a function of wavelength and delay time; c and d) Decay associated TA spectra recorded at different delay times calculated by singular value decomposition analysis; e and f) Normalized PIA decay dynamics of fs‐TA signals.

To get more deeper insight into the photoexcited charge carrier dynamics, femtosecond transient absorption (fs‐TA) spectrum was measured for W‐ and G‐[DADPA]PbBr_5_ as example. The wavelength and delay time‐dependent pseudocolor TA plots are depicted in **Figure** **4**a,b. Upon excitation of 310 nm pulsed laser, broad positive photo‐induced absorption (PIA) plateau is emerged in probe region (400–700 nm) for both W‐ and G‐[DADPA]PbBr_5_ demonstrating the direct evidence of charge carrier relaxation process. Significantly, the stronger positive absorption of G‐[DADPA]PbBr_5_ than W‐[DADPA]PbBr_5_ demonstrates the higher concentration of photo‐induced charge carriers in the former (Figure 4c,d). As shown in Figure S16 (Supporting Information), the formation time (120 and 235 fs) of excited electrons were determined based on the raising time of pump‐induced absorption at different excitation wavelengths covering 400–700 nm, respectively, indicating that charge carriers are more rapidly formed in W‐[DADPA]PbBr_5_. Meanwhile, the almost identical PIA rise times at different wavelengths indicate that the charge carrier can be formed synchronously with the same excited state. The intensities of TA absorption signal gradually decrease with increasing of probe delay times, which is similar to most of metal halide perovskites.^[^ ^31^ ^]^ The fs‐TA dynamics were analyzed by means of the global fitting, and the results are depicted in Figure 4e,f. The TA dynamic of W‐[DADPA]PbBr_5_ can be decomposed into three time‐resolved components including ultrafast lifetime of *τ*_1_ = 3.7 ps, middle fast lifetime of *τ*_2_ = 191.7 ps, and a slow lifetime of *τ*_3_ > 0.621 ns. Similarly, the TA dynamic of G‐[DADPA]PbBr_5_ also contains three components of *τ*_1_: 3.8 ps (ultrafast), *τ*_2_: 238.3 ps (middle), and *τ*_3_: 1.288 ns (slow). The ultrafast component (*τ*_1_) of W‐ and G‐[DADPA]PbBr_5_ can be assigned to the cooling process of hot charge carriers, which is generally a fast process on order of picosecond in semiconducting materials. The middle PIA decay process belongs to the *V*_Br_ defect trapping process corresponding to the absorption tail, which is similar to most of double perovskite nanocrystals. The slow decay signal represents the lifetime of band‐edge free charge carriers. Comparing with W‐[DADPA]PbBr_5_, the longer average lifetime of G‐[DADPA]PbBr_5_ reflects the slower relaxation dynamics. Overall, the relaxation manner of fs‐TA dynamics basically agrees with the time‐resolved PL studies for both perovskites.

**Figure 5:**
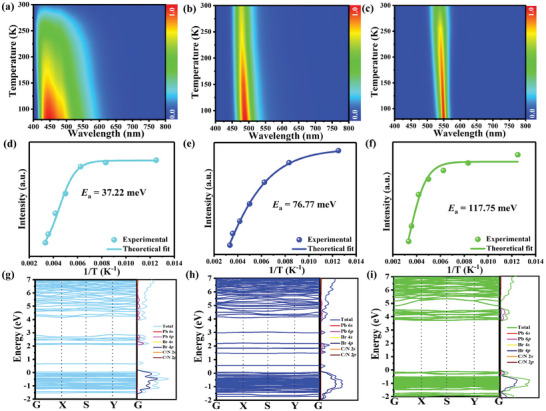
a–c) 3D color maps of PL emission at varied temperatures for W‐, B‐ and G‐[DADPA]PbBr_5_; d–f) experimental and fitted emission intensity versus reciprocal temperature for W‐, B‐ and G‐[DADPA]PbBr_5_; g–i) calculated band structure and DOS of W‐, B‐ and G‐[DADPA]PbBr_5_.

Temperature‐dependent PL emission spectrum was further conducted to in‐depth understand the nature of distinct luminescent performance. As shown in **Figure** **5**a–c, the PL emission spectra show identical spectral profiles without additional splitting emission peaks ruling out the possibility of multiple emitting channels. With the temperature decreasing, the maximum emission peaks show a slight red‐shift for all W‐, B‐, and G‐[DADPA]PbBr_5_ (Figure S17, Supporting Information). The shift toward lower energy is mainly determined by the thermal expansion interaction based on the theoretical fitting of the temperature‐dependent evolution of emission energy (Figure S18, Supporting Information). At the same time, all the emission intensities of three samples monotonously increase with temperature decreasing owing to the weakened thermal vibration associated nonradiative recombination process. Accordingly, the thermal activation energy was calculated to evaluate the energy barrier between radiative and nonradiative states by fitting the temperature‐dependent PL intensity based on Arrhenius Equation (4) (Figure 5d–f):

(4) $$I\left(T\right)=\frac{I_{0}}{1+A\exp\left(-\frac{E_{b}}{k_{B}T}\right)}$$

where *I*_PL_ and *I*_0_ represent the integrated emission intensity at different temperatures (*T*) and 0 K, respectively, *E*_b_ belongs to energy of exciton binding and *k*_B_ is the Boltzmann constant. The calculated *E*_b_ values follow the order of W‐[DADPA]PbBr_5_ (37.22 meV) < B‐[DADPA]PbBr_5_ (76.77 meV) < G‐[DADPA]PbBr_5_ (117.75 meV), which is in accordance with the variation tendency of PLQY. That is, the larger thermal activation energy is favorable for stronger radiative recombination and higher emission efficiency, further manifesting the important regulating effect of controllable *V*_Br_. To get insight into the influence of structural defects on the multiple adjustable PL performance, first‐principles calculations were further performed for three structural modes with different *V*_Br_ densities based on DFT (Figure S19, Supporting Information). As shown in Figure 5g–i, the band gap slightly decreases from 4.12 to 3.83 eV with reducing the *V*_Br_ content from W‐ to G‐[DADPA]PbBr_5_, which is in accordance with the decreased emission energy. Besides, all the valence band maximums (VBMs) and conduction band minimums (CBMs) of are located at the same symmetry point of W‐, B‐ and G‐[DADPA]PbBr_5_, indicating all equipped with direct band gaps, according to the experimental results obtained from Tauc′s plot analysis. The states around the Fermi level mainly originate from the mixed bands of Pb‐6*s*6*p* and Br‐4*p* orbitals, while the contribution of organic cation is negligible, which suggests that the photophysical behaviors are mainly dominated by the [Pb_2_Br_10_]^6−^ dimer. In addition, the *V*_Br_ gives rise to a large amount of isolated narrow in‐gap states in the band gap for W‐ and B‐[DADPA]PbBr_5_, while there is not any sub‐band state in the electronic band structure of G‐[DADPA]PbBr_5_. These sub‐band states not only consume the excited electrons but also readily trap partial charge carriers as new radiative centers.

**Figure 6:**
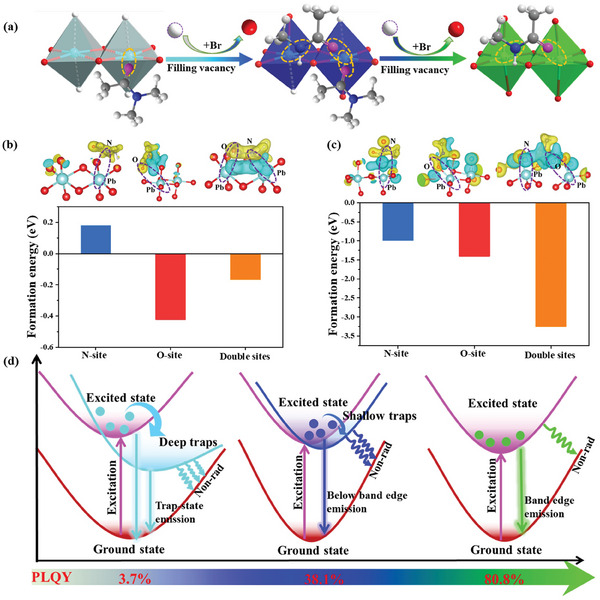
a) The simulated adsorption models of DMA and NMA molecule; b,c) charge density difference and adsorption formation energies of DMA b) and NMA c) molecule on the [DADPA]PbBr_5_ perovskite surface via N‐, O‐ and double N‐, O‐band connections. d) Schematic illustration of the possible photophysical mechanism for the W‐, B‐, and G‐[DADPA]PbBr_5_.

Based on the above systematical experimental investigations and theoretical calculations, the underlying mechanism of diversified PL performance in [DADPA]PbBr_5_ family is proposed as illustrated in **Figure** **6**. In the wet‐chemistry synthesis process of metal halide perovskites, the halogen vacancies are unavoidably formed due to the appearance of abundant organic molecules or ligands, which compete with halide ions to directly coordinate the lead atom or bind with halogen atom through hydrogen bonds to weak the lead halogen interaction, resulting in inevitable halogen vacancies.^[^ ^32^ ^]^ The halogen vacancies would become nonradiative centers that are capable of degrading the optical absorption and luminescent performance of perovskite materials.^[^ ^33^ ^]^ In this work, we adopt the similar DMA and NMA molecules as a pair of organic ligands, which possess slightly different coordination abilities to Pb^2+^ ions and hydrogen bonding interactions to Br^−^ ions (Figure 6a).

To unveil the different passivation effect, the adsorption models and corresponding formation energies of DMA and NMA molecules on the surface of [Pb_2_Br_10_]^6−^ dimers were investigated including N‐site, O‐site and double N‐, O‐site types of connection models (Figure S20, Supporting Information)^[^ ^34^ ^]^ As shown in Figure 6b,c, all the formation energies of Pb─O bonds are larger than those of Pb─N bonds in both DMA and NMA molecules, indicating higher binding force of Pb─O bonds. The formation energy of Pb─N bonds in DMA is positive, which indicates that such a connection is almost impossible, mainly due to the large steric hindrance of two methyl groups in DMA molecules. By contrast, the negative formation energy of Pb─N bonds between DMA and [DADPA]PbBr_5_ demonstrates the forceful coordination ability. Especially, the formation energies of double O‐, N‐site types of connecting manners for NMA are larger than those of DMA molecule, suggesting a high probability for the formation of Pb─O and Pb─N bonds between NMA and [DADPA]PbBr_5_. Furthermore, the adsorption of NMA molecule on the surface of [DADPA]PbBr_5_ gives rise to more hydrogen bonds than DMA, which also reinforce the interaction ability of NMA toward [DADPA]PbBr_5_ (Figures S21 and S22, Supporting Information). Therefore, the NMA molecule is easily adsorbed on the surface of [DADPA]PbBr_5_ bonding with Pb atoms through strong interactions, which stabilize the [PbBr_6_] octahedron and reduce the *V*_Br_ density.

Through increasing the relative ratio of NMA: DMA to regulate the anchoring interactions and passivation effects, the surface defects are patched and content of *V*_Br_ is rationally reduced until it achieves perfect crystal structure with ideal chemical composition. When the bromine vacancy density is too high in the crystal structure, the *V*_Br_ defect forms impurity energy levels in the sub‐bandgap acting as deep traps (Figure 6d). Most of the excited carriers are consumed by these deep states and then return to the ground states through multi‐phonon scattering as nonradiative recombination process. Furthermore, small quantity of excited carriers undergoes radiative transition from conduction bands or deep trap states to ground states, generating weak broadband light emission due to the inhomogeneous broadening induced by random spatial distributions of *V*_Br_ in crystal lattice. With the significant decrease in *V*_Br_ density, the defect energy levels are located just below the conduction band as shallow traps. Most of excited electrons on conduction bands directly return to the ground state as radiative recombination process to form narrow blue band edge emission, and other carriers are trapped by the shallow states as nonradiative recombination, which weakens the emission efficiency. The less *V*_Br_ locates in the crystal lattice, the more efficient radiative recombination and emission efficiency is obtained for narrow band edge emission. Therefore, the PL emission wavelength finally red‐shifts from 445 to 535 nm and PLQY gradually increases from 3.7% to 80.8% as narrow green light emission in the case of least *V*_Br_ content and band gap. In brief, the tailorable *V*_Br_ content realizes the different kinds of trap states in the sub‐bandgap of [DADPA]PbBr_5_, which gives rise to adjustable band gap, radiative recombination mechanism, and PL emission performance.

Considering that crystallization environment plays a crucial role in the *V*_Br_ density and PL property of [DADPA]PbBr_5_, we further analyzed the emission stabilities of W‐, B‐ and G‐[DADPA]PbBr_5_ in various physical and chemical environments. After continuous storing in humid air over one month, under irradiation of strong UV light or soaking in various organic solvents over long time, the same PXRD patterns and emission spectral profiles indicate the stable crystal structure and PL property with slightly reduced emission intensities (Figures S23–S31, Supporting Information). Furthermore, the thermogravimetric analysis (TGA) curves show the sample can be stable up to ≈300 °C without structural decomposition, and PXRD patterns and PL emission spectra demonstrate the stable structural and emitting performance under continuous heating from room temperature to 160 °C, showcasing the application potential at high‐temperature environment (Figures S32–S35, Supporting Information). Benefiting from the diversified and stable PL properties, this [DADPA]PbBr_5_ family can be used to fabricate various LED devices. By directly coating the powders of W‐, B‐ and G‐[DADPA]PbBr_5_ samples on 365 nm UV chip, the as‐fabricated LEDs display bright and stable white, blue, and green light at drive current of 20 mA, respectively, indicating the promising application prospects in solid state lighting installations (Figures S36–S38, Supporting Information). It is known to all that an ultrapure green light emitter with a narrow emission range (525–535 nm) and FWHM < 25 nm is critical for backlit LED in liquid‐crystal display (LCD) according to the Rec. 2020 standard. Herein, the G‐[DADPA]PbBr_5_ SC displays ultra narrow green light emission at 535 nm with FWHM of 28 nm, and the color purity is calculated to be 92.74%, which is significantly higher than all the previously reported green emissive low‐dimensional OIHPs, such as [bmpy]_6_Pb_3_Br_12_ (20.31%), [bmpy]_9_[MnBr_4_]_2_[Pb_3_Br_11_] (82.73%), [bmpy]_9_[ZnBr_4_]_2_[Pb_3_Br_11_] (79.60%) and (bmpy)_2_MnBr_4_ (79.23%), etc (Table S1, Supporting Information).^[^ ^22^, ^35^ ^]^ The CIE chromaticity coordinate of G‐[DADPA]PbBr_5_ (0.23, 0.73) is close to 3D MAPbBr_3_ nanocrystals and approximately achieves the Rec. 2020 standard representing one of the purest green light emission among all the low‐dimensional OIHPs to our best knowledge (Figure S39a, Supporting Information).^[^ ^36^ ^]^ To explore the potential application in solid‐state display devices, we fabricate the LED based on G‐[DADPA]PbBr_5_ and commercial red phosphor K_2_SiF_6_:Mn^4+^ on a 455 nm blue LED chip. The calculated color gamut covers the color space of 132.7%, 94.0%, and 70.2% of ITU‐R BT.709 standard, NTSC, and Rec. 2020 standard in CIE‐1931 diagram,^[^ ^37^ ^]^ respectively, indicating the significant application prospects in new‐generation LCD technology (Figure S39b,c, Supporting Information).^[^ ^38^ ^]^

**Figure 7:**
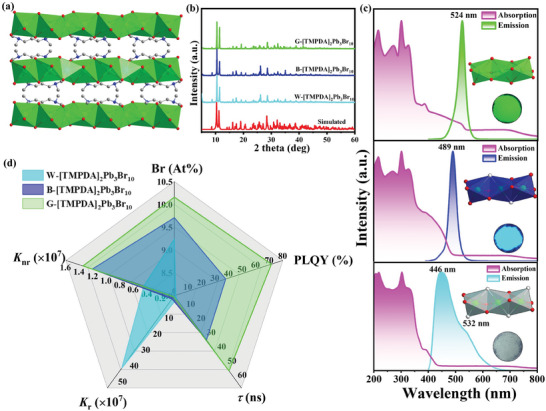
Structural and PL characterizations of [TMPDA]_2_Pb_3_Br_10_ family: a) The packing structure based on [TMPDA]^2+^ cation and 1D [Pb_3_Br_10_]^4−^ chain; b) powder X‐ray diffraction patterns; c) absorption and emission spectra (inset: structural diagram and photo images under UV light); d) radar map of comparisons for *λ*_em_ (nm), FWHM (nm), PLQY (%), time‐resolved PL decay (ns), radiative (*K*_r_) and nonradiative (*K*_nr_) recombination rates of W‐, B‐ and G‐ [TMPDA]_2_Pb_3_Br_10_.

More significantly, such a *V*_Br_ tuning strategy of adjustable PL performance can be extended to other low‐dimensional OIHPs as exemplified using [TMPDA]_2_Pb_3_Br_10_ (TMPDA = tetramethyl‐1,3‐diaminopropane).^[^ ^39^ ^]^ The single crystals of [TMPDA]_2_Pb_3_Br_10_ were prepared by using the solvothermal reaction and the structure is based on 1D [Pb_3_Br_10_]^4−^ chain surrounded by [TMPDA]^2+^ cations (**Figure** **7**a). Through fine‐tuning the crystallization environment, [TMPDA]_2_Pb_3_Br_10_ SCs can also display adjustable PL properties from broadband white to narrow green light emission upon irradiation of a 365 nm UV lamp. Herein, the white, blue, and green emissive [TMPDA]_2_Pb_3_Br_10_ SCs were taken as examples to manifest the *V*_Br_ density‐dependent PL performance. Single crystal and powder X‐ray diffraction reveal the same crystal structure of these samples but with the different Pb: Br ratios of 3: 9.24, 3: 9.71, and 3: 10.16, respectively, for W‐, B‐ and G‐[TMPDA]_2_Pb_3_Br_10_ based on ICP and EDS investigation results indicating the gradually decreased *V*_Br_ content (Figure 7b; Table S2, Supporting Information). In addition, the photo energy‐dependent absorption tail at low energy also indicates the gradually decreased *V*_Br_ content from W‐, B‐, and G‐[TMPDA]_2_Pb_3_Br_10_ (Figure 7c; Figures S40 and S41, Supporting Information). Detailed PL spectroscopy gives the dominant emission wavelength (*λ*_em_) at 446, 489, and 524 nm with gradually narrowed FWHM of 90, 30, and 25 nm for W‐, B‐ and G‐[TMPDA]_2_Pb_3_Br_10_, respectively (Figure 7c). As shown in the radar map of Figure 7d, the representative properties of W‐, B‐ and G‐[TMPDA]_2_Pb_3_Br_10_ were concisely compared including *λ*_em_, FWHM, PLQY, lifetime, *K*_r,_ and *K*_nr_ based on varied *V*_Br_ content. The PLQY significantly increases from 0.98% to 37.85% and 71.59%, and lifetime extends from 2.18 to 31.24 ns and 53.03 ns (Figures S42 and S43 and Table S3, Supporting Information). With the decreasing of *V*_Br_ from W‐, B‐ to G‐[TMPDA]_2_Pb_3_Br_10_, the *K*_r_ gradually increases while *K*_nr_ decreases step by step indicating the enhanced radiative recombination rate. All these results clearly demonstrate that the accurate manipulating of *V*_Br_ content successfully realizes adjustable PL properties including varied emission wavelength, linewidth, PLQY, and lifetime in the same structural platform of [TMPDA]_2_Pb_3_Br_10_. In this context, such halogen vacancy tuning strategy can be universally applied in other low‐dimensional OIHPs, which provides a novel proof‐of‐concept property optimization strategy and enriches perovskite chemistry.

## 3 Conclusion

In this work, a defect‐tuning strategy was proposed for the first time and validated to manipulate the photophysical properties of halide perovskites on multiple dimensions. Specifically, the finely‐tuned crystallization environment successfully realized the controllable concentration of halogen vacancies, which gives rise to tailorable luminescent properties from broad trap‐state emission to narrow band‐edge emission with enhanced PLQY and color purity due to suppressed nonradiative recombination process. This achievement highlights a proof‐of‐concept luminescent modification strategy expanding the scientific horizon of luminescent perovskite materials. First, the density of halogen vacancies is accurately controlled over a wide range based on the same structural platform of OIHP SCs, which provides a new opportunity to finely tune the chemical composition of perovskites while retaining the parent lattice structure. Secondly, the combined spectroscopy and theoretical studies demonstrate that halogen vacancies play a crucial role in the band structures and carrier transition pathway, which results in diversified PL properties with multiple adjustable PL properties including emission spectral range, linewidth, lifetime, and PLQY. Remarkably, this vacancy tuning strategy toward low‐dimensional perovskites is superior to the conventional post‐synthetic surface passivation method that merely improves the emission efficiency of perovskite nanocrystals. Therefore, this work not only provides a novel functional modification strategy but also deepens the understanding on defect‐property relationship in perovskite materials. Unlike the bandgap‐determined emission of 3D PNCs, the halogen vacancy‐induced adjustable emission is unprecedented in perovskite chemistry. This breakthrough is comparable to conventional defect engineering in inorganic semiconducting materials, such as ZnO, GaN, and ZnSe, positioning perovskite hybrids as more versatile semiconductors with outstanding photophysical properties and potentials for cutting‐edge optoelectronic applications. In this regard, we believe this vacancy fine‐tuning strategy paves a novel window to rationally design luminescent perovskites on the atomic‐precise level.

## Conflict of Interest

The authors declare no conflict of interest.

## Supporting information



Supporting Information

Supporting Information

## Data Availability

The data that support the findings of this study are available from the corresponding author upon reasonable request.
