# Supplementary material for: In Situ Halide Vacancy Tuning of Low‐Dimensional Lead Perovskites to Realize Multiple Adjustable Luminescence Performance
Source: Adv Sci (Weinh). 2025 Mar 17;12(18):2412459. doi: 10.1002/advs.202412459 (PMC12079511; doi:10.1002/advs.202412459)
Supplement: Supplementary file 1 — Supporting Information [file ADVS-12-2412459-s001.docx]

**Supplementary Information**

***In-situ* Halide Vacancy Tuning of Low-Dimensional Lead Perovskites to Realize Multiple Adjustable Luminescence Performance**

Chen Sun,^[a,b]^ Chang-Qing Jing,^[a,c]^ Dong-Yang Li,^[a,b]^ Meng-Han Dong,^[a]^ Ming-Xue An,^[a]^ Zhong-Hui Zhang,^[a]^ Cheng-Yang Yue,*^[a]^ Honghan Fei,*^[b]^ and Xiao-Wu Lei*^[a]^

[a] C. Sun, C. Q. Jing, D. Y. Li, Prof. C.Y. Yue, Prof. X. W. Lei
School of Chemistry, Chemical Engineering and Materials, Jining University, Qufu, Shandong, 273155, China

[b] C. Sun, D. Y. Li, Prof. H. H. Fei

Shanghai Key Laboratory of Chemical Assessment and Sustain ability, School of Chemical Science and Engineering, Tongji University, Shanghai 200092, China

[c] C. Q. Jing

Jiangsu Key Laboratory for Science and Applications of Molecular Ferroelectrics and School of Chemistry and Chemical Engineering Southeast University, Nanjing 211189, China

*Corresponding authors: Cheng-Yang Yue; Honghan Fei; Xiao-Wu Lei

**E-mail addresses:** yuecy@jnxy.edu.cn (C.-Y. Yue); fei@tongji.edu.cn (H. Fei); leixw@jnxy.edu.cn (X.-W. Lei)

**Experimental Section**

***Materials and methods***

All chemicals were purchased from commercial sources and directly used in the preparation reaction without further purification. PbBr_2_ (99.99%), 3,3'-Diaminodipropylamine (DADPA, C_6_H_17_N_3_, 98%), N-Methylacetamide (NMA, C_3_H_7_NO, 99%), N,N-Dimethylacetamide (DMA, C_4_H_9_NO, 99%), methanol (MeOH, 99%), ethanol (EtOH, 99%), tetramethyl-1,3-diaminopropane (TMPDA, 99.0%), N-methylformamide (NMF, 99.5%), N,N-Dimethylformamide (DMF), hydrobromic acid (HBr, 40%).

***Synthesis of*** ***W-[DADPA]PbBr_5_:*** First, 0.1101 g of PbBr_2_ (0.3 mmol) was dissolved in a mixed solution of HBr solution (1 mL), methanol (3 mL) and DMA (3mL), then 0.078 g of DADPA (0.6 mmol) was added into the solution to form a pale white precipitate. The above precipitate was transferred into a 25 mL dry flask and refluxed at 140 °C for 48 h. Subsequently, the colorless crystals were isolated after vacuum filtration and washed with ethanol, in yield of 72% based on PbBr_2_, and determined as C_6_H_20_N_3_PbBr_5_ by single-crystal X-ray diffraction.

***Synthesis of G-[DADPA]PbBr_5_:*** Light yellow crystals of G-[DADPA]PbBr_5_ can be synthesized in the same manner as for W-[DADPA]PbBr_5_ but with NMA (3 mL) instead of DMA. Yield 68% (based on PbBr_2_).

***Synthesis of B-[DADPA]PbBr_5_ and*** ***intermediate phases:***Serial intermediate phases were prepared via the same reaction methods except rationally manage the volume radio of DMA and NMA, and the DMA/NMA volume ratio was continuously controlled from 5:1 to 1:6. Here, the light green crystals of B-[DADPA]PbBr_5_ were prepared when the volume ratio of DMA and NMA was 1:1. Yield 66% based on PbBr_2_.

***Synthesis of W-[TMPDA]_2_Pb_3_Br_10_.*** First, 0.1101 g of PbBr_2_ (0.3 mmol) was dissolved in a mixed solution of HBr solution (1 mL), EtOH (2 mL) and DMF (3mL), then 0.100 g of TMPDA (0.5 mmol) was added into the solution to form a pale white precipitate. The suspension was constantly stirred about half an hour and then transfer into a 25 mL dry flask, which was then sealed and heated at constant temperature of 140 °C at least three days. After the reaction, lots of colorless plane-like crystals were filtrated and washed with ethanol three times in yield of 61% based on PbBr_2_, and determined as C_14_H_40_N_4_Pb_3_Br_10_ by single-crystal X-ray diffraction.

***Synthesis of G-[TMPDA]_2_Pb_3_Br_10_***. Light yellow crystals of G-[TMPDA]_2_Pb_3_Br_10_ can be synthesized in the same manner as for W-[TMPDA]_2_Pb_3_Br_10_ but with NMF (3 mL) instead of DMF with yield of 80% based on PbBr_2_.

***Synthesis of B-[TMPDA]_2_Pb_3_Br_10_.*** Serial intermediate phases were prepared via the same reaction methods except rationally manage the volume radio of DMF and NMF. Here, the light green crystals of B-[TMPDA]_2_Pb_3_Br_10_ were chosen when the volume ratio of DMF and NMF was 1:1 in yield of 66% based on PbBr_2_.

***Single Crystal X-ray Diffraction.*** A suitable size single crystal was mounted onto a glass fiber. The single-crystal data of compounds W-[DADPA]PbBr_5_, B-[DADPA]PbBr_5_ and G-[DADPA]PbBr_5_ were collected on the Bruker Apex II CCD diffractometer with Mo-K*α* radiation (λ = 0.71073 Å) at 298(2) K. The structure was solved by direct methods and expanded routinely. The model was refined by full-matrix least-squares analysis of *F*^2^ against all reflections using SHELXTL-2018 program. All non-hydrogen atoms were refined with anisotropic thermal displacement parameters, and hydrogen atoms of organic molecules were positioned geometrically and refined isotropically. Structural refinement parameters of W-[DADPA]PbBr_5_, B-[DADPA]PbBr_5_ and G-[DADPA]PbBr_5_ were summarized in Table S4 and important bond parameters were listed in Table S5-S9. The X-ray crystallographic data were deposited at the Cambridge Crystallographic Data Centre (CCDC), under deposition numbers CCDC 2259995-2259997 for W-[DADPA]PbBr_5_, B-[DADPA]PbBr_5_ and G-[DADPA]PbBr_5_, containing the supplementary crystallographic data for this paper. These data can be obtained free of charge from https://www.ccdc.cam.ac.uk/structures/.

***Characterizations.*** The powder X-ray diffraction (PXRD) analysis was performed on Bruker D2 powder X-ray diffractometer equipped with Cu-Kα radiation at a voltage of 30 kV and a current of 10 mA. Fourier-transform Infrared (FT-IR) spectra were recorded using a BRUKER ALPHA spectrophotometer in 4000~400 cm^-1^ region with a resolution of 2 cm^-1^. Thermogravimetric analysis (TGA) was carried out using a TGA Q500 differential thermal analyzer. The samples were heated from room temperature to 800 °C with a heating rate of 10 °C/min under N_2_ stream (60 mL/min). The microstructure and elemental mapping were characterized on TESCAN MIRA LMS Xplore field-emission scanning electron microscope (SEM) equipped with an energy-dispersive X-ray spectrometer. The X-ray photoelectron spectroscopy (XPS) spectra were tested on the Thermo Fisher ESCALAB Xi+ instrument, USA. Inductively coupled plasma-Mass Spectrometry (ICP-MS) was performed on Agilent 7800. The Raman spectrum measurement was performed on the powder sample in the range of 50-4000 cm^-1^ by using Horiba Scientific LabRam HR Evolution under 365 nm excitation wavelength.

***UV-vis absorption optical spectra Characterization***. The solid-state UV-vis absorption optical spectrum in the range of 200-800 nm wavelength was collected at room temperature by a PE Lambda 900 UV/Vis spectrophotometer equipped with an integrating sphere. All test samples were grinded into *μ*m-sized powders, then packed into the sample cell and BaSO_4_ (100%) was used as a reference standard. The Kubelka-Munk plots {F[(F(*α*)*hν*]}^1/2^ and {F[(F(*α*)*hν*]}^2^ as functions of photon energy (*hν*) are applied to the UV-Vis absorption spectra. The optical absorption coefficient, *F*(*α*) = *A*^2^/2(1-*A*), is calculated where *A* is absorbance, *h* is the Planck constant, and *ν* is the frequency of light at a specific wavelength. The extrapolation of linear region affords the bandgap value of W-, B-and G-[DADPA]PbBr_5_.

***Photoluminescence Property*** ***Characterization.*** The PL spectra were performed on an Edinbergh FLS1000 fluorescence spectrometer. The photoluminescence quantum yield (PLQY) was achieved by incorporating an integrating sphere into the FL1000 spectrofluorometer. The PLQY was calculated based on the equation: *ƞ*_QE_ = *I*_S_/(*E*_R_**-***E*_S_), where *I*_S_ represents the luminescence emission spectrum of the sample, *E*_R_ is the spectrum of the excitation light from the empty integrated sphere (without the sample), and *E*_S_ is the excitation spectrum for exciting the sample. The time-resolved decay data were carried out using the Edinburgh FLS1000 spectrofluorometer with a picosecond pulsed diode laser. The average lifetime was obtained by exponential fitting according to the following equation:

$$\tau_{\mathrm{avg}}=\frac{\sum a_{i}\tau_{i}^{2}}{\sum a_{i}\tau_{i}} i=1,2$$

where 𝑎_𝑖_ represents the amplitude of each component and 𝜏_𝑖_ represents the decay time. The corresponding Commission Internationale Eclairage (CIE) chromaticity coordinates were calculated based on PL emission spectrum.

***Ultrafast transient absorption (TA) spectroscopy.*** TA spectroscopy was performed at room temperature by a regenerative amplified Ti: sapphire laser system (Coherent; 800 nm, 35 fs, 6 mJ/pulse, and 1 kHz repetition rate), nonlinear frequency mixing techniques and the Helios spectrometer (Ultrafast Systems LLC). The 800 nm output pulse from the regenerative amplifier was split into two parts using a 50% beam splitter. The transmission part was used to pump the TOPAS optical parametric amplifier, producing a 310 nm pump beam. The reflecting part was attenuated by a neutral density filter and focused into a 2 mm thick CaF_2_ window, producing a continuous white light of 350 nm to 650 nm as the probe beam. The probe beam was then focused onto the sample with a parabolic reflector. Then, the probe beam was collimated and focused into a fiber-coupled spectrometer and detected at a frequency of 1 KHz. The delay between the pump and probe pulses was controlled by a motorized delay stage. The pump pulses were chopped by a synchronized chopper at 500 Hz and the absorbance change was calculated with two adjacent probe pulses (pump-blocked and pump-unblocked).

***The calculation of color purity (CP)*.** The color purity or saturation of a specific light source is defined by the percentage of the linear distance between the CIE coordinates (*x*, *y*) of the target emission and CIE-1931 equal-energy point (*x_i_* = 0.33, *y_i_* = 0.33) to the linear distance between the CIE coordinates of corresponding dominant-wavelength point (*x_d_*, *y_d_*) and equal-energy point (0.33, 0.33). Specifically, the color purity can be calculated by the following equation:

$$Color purity= \frac{\sqrt{{{(x-x_{i})}^{2}+(y-y_{i})}^{2}}}{\sqrt{{{(x_{d}-x_{i})}^{2}+(y_{d}-y_{i})}^{2}}}\times100\%$$

the green light emission of G-[DADPA]PbBr_5_ corresponds to the CIE chromaticity coordinates of (*x*, *y*) = (0.23, 0.73) and dominant wavelength of 535 nm with CIE coordinates of (*x_d_*, *y_d_*) = (0.22, 0.76).

***LED Fabrication.*** To investigate the potential application in solid-state lighting diode, the single W-[DADPA]PbBr_5_, B-[DADPA]PbBr_5_ or G-[DADPA]PbBr_5_ sample was respectively coated on a UV chip (*λ*_em_ = 365 nm) to fabricate the white, blue or green LED. The photoelectric properties including the luminous efficiency, EL spectrum, CCT, CIE color coordinates and color rendering index (CRI) of the LED were measured by using an integrating sphere spectroradiometer. In addition, to explore the potential application in solid-state display device, mixture of green phosphor G-[DADPA]PbBr_5_ and red phosphor KSF:Mn^4+^ was coated on a blue InGaN chip (*λ*_em_ = 455 nm) to fabricate a LED considering the narrow FWHM of G-[DADPA]PbBr_5_. The color gamut of LED was calculated based on the CIE coordinates of G-[DADPA]PbBr_5_, KSF:Mn^4+^ phosphors and InGaN chip.

***Theoretical Calculation on Electronic Band Structure.*** All calculations were carried out for the material in the framework of Density Functional Theory (DFT) using the Vienna Ab initio Simulation Package (VASP 6.3.0).^[1-3]^ The generalised gradient approximation (GGA) of the Perdew-Burke-Ernzerhof (PBE) function was used to describe the exchange-correlation energy.^[4]^ The projected augmented wave (PAW) method and pseudopotentials were used to describe the interactions between valence electrons and ions.^[5]^ To ensure the efficiency of the computational results and parallel computing. A 2×2×2 *k*-point grid under Monkhorst-Pack is used in the optimisation process and 450 eV truncation energy is set. Partial occupancies of the Kohn-Sham orbitals were allowed using the Gaussian smearing method and a width of 0.05 eV. A 1×1×1 of original crystal cell was built with different occupancy of each Br atom (96%, 98% and 100%) as the molecular structure models for W-[DADPA]PbBr_5_, B-[DADPA]PbBr_5_ and G-[DADPA]PbBr_5_ based on the ICP investigation results. The lattice parameters and ionic positions of all crystals were fully relaxed, and the convergence criteria for the total energy of all relaxed atoms and the final force were 10^-5^ eV and 0.03 eV/Å, respectively.

***Theoretical Simulation of Structure and Calculation of Adsorption Enthalpy.*** The adsorption energy (*E*_ads_) for each elemental step is defined as:

$$E_{ads}=E_{AB}-E_{A}-E_{B}$$

where *E*_AB_ is the energy of composite structure, *E*_A_ represents the energy of adsorption surface, *E*_B_ represents the adsorbate energy based on density functional theory calculations and the zero-point energy correction, respectively.


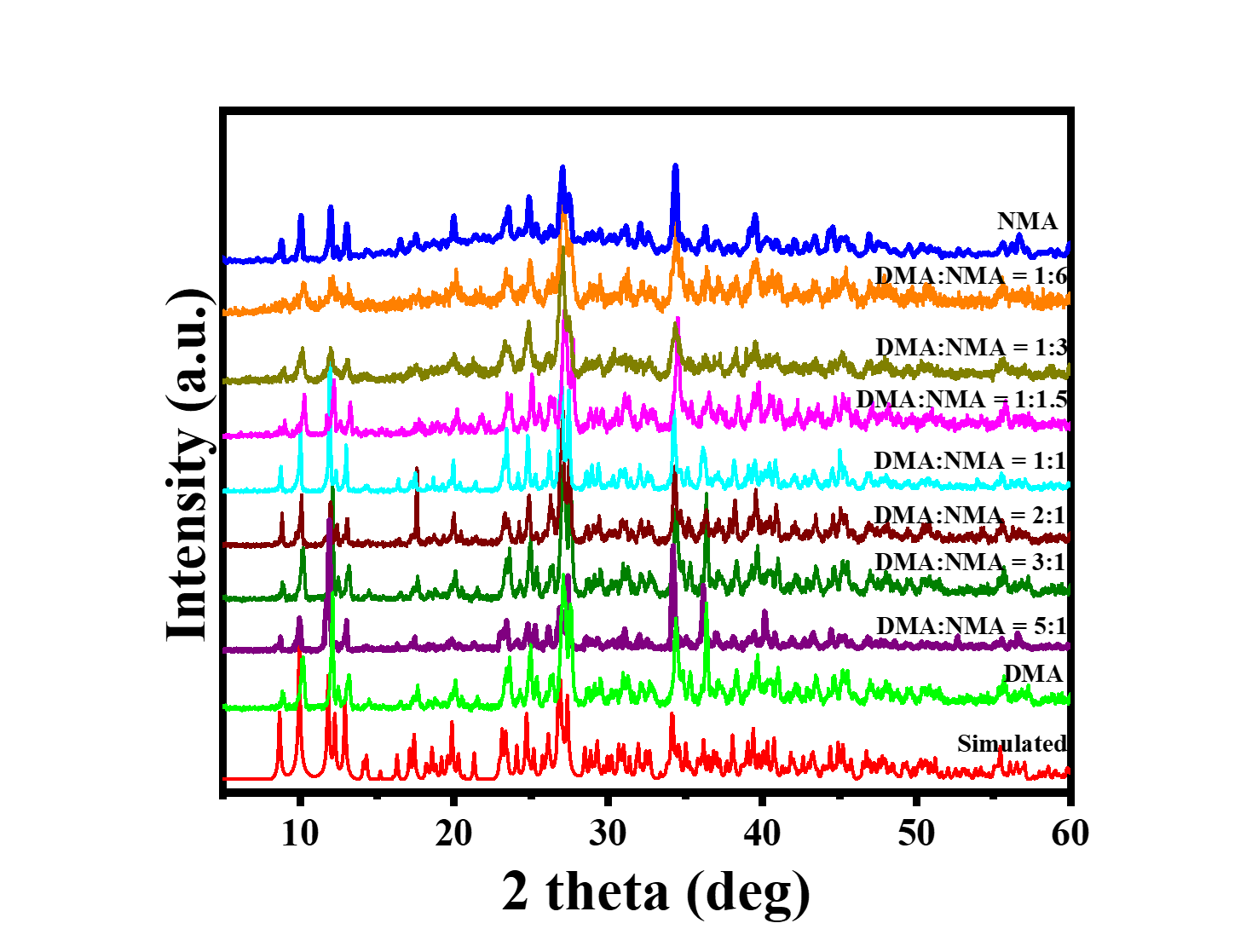


**Figure S1.** The experimental and simulated PXRD patterns of as-synthesized serial intermediate phases from W-[DADPA]PbBr_5_ to G-[DADPA]PbBr_5_.

**Figure S2.** IR spectra of W-[DADPA]PbBr_5_, B-[DADPA]PbBr_5_ and G-[DADPA]PbBr_5_.

**Figure S3.** Raman spectra of W-[DADPA]PbBr_5_, B-[DADPA]PbBr_5_ and G-[DADPA]PbBr_5_ at room temperature.


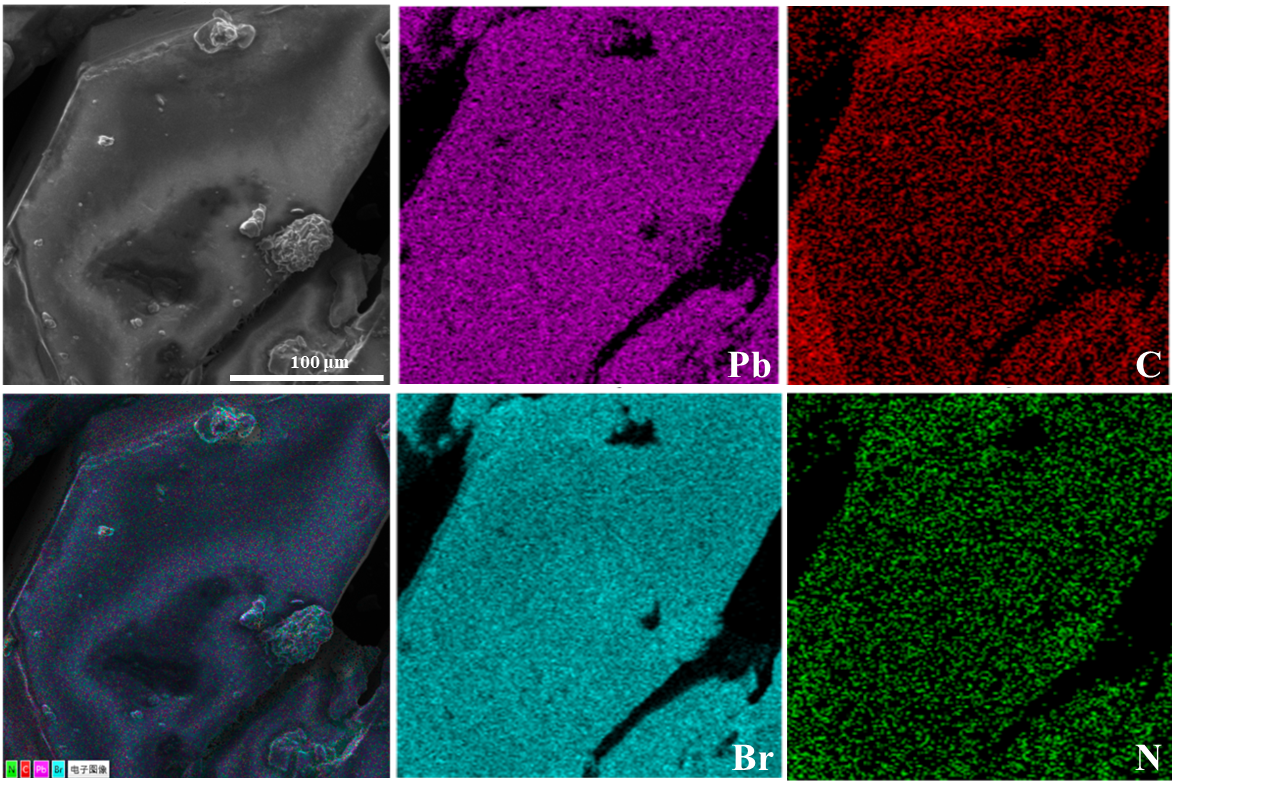
(a)


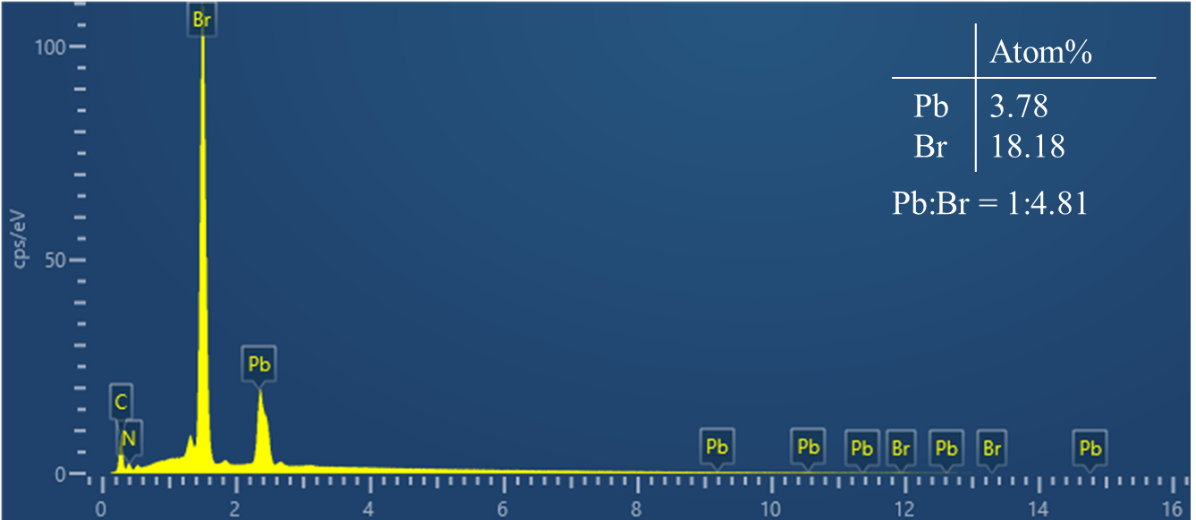
 (b)

**Figure S4**. The SEM image and elemental mapping images of Pb, Br, C and N elements (a), EDX analysis result (b) of W-[DADPA]PbBr_5_.


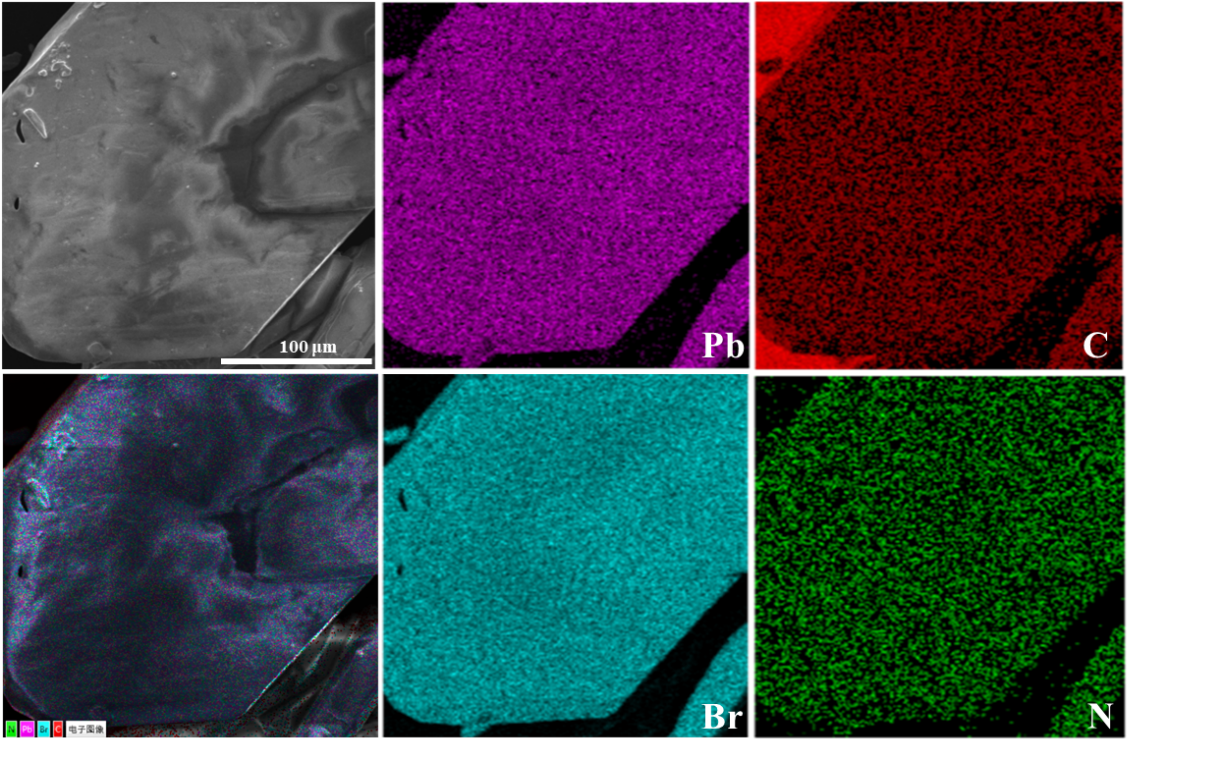
 (a)


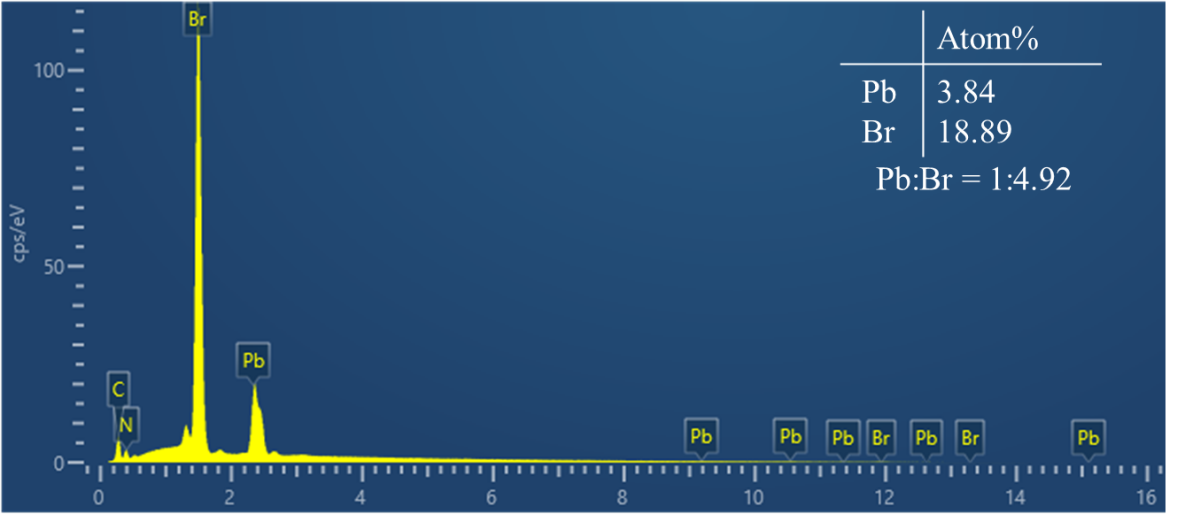
 (b)

**Figure S5**. The SEM image and elemental mapping images of Pb, Br, C and N elements (a), EDX analysis result (b) of B-[DADPA]PbBr_5_.


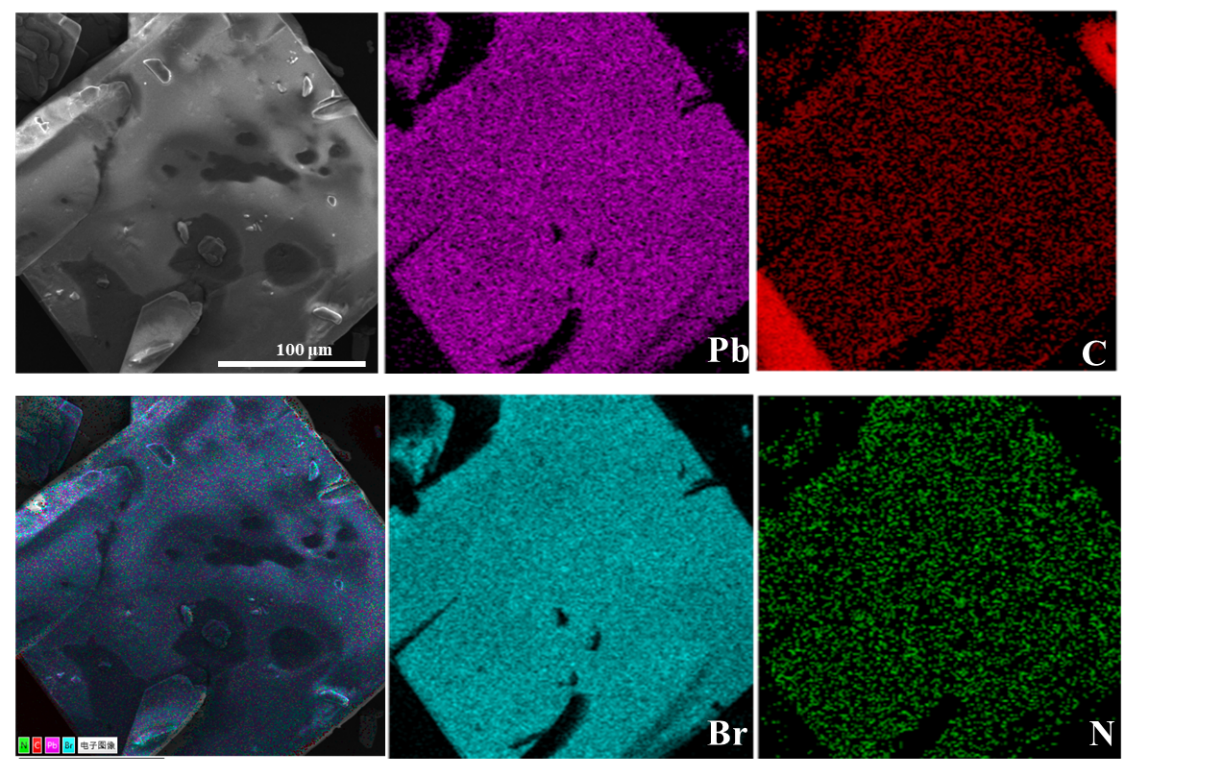
 (a)


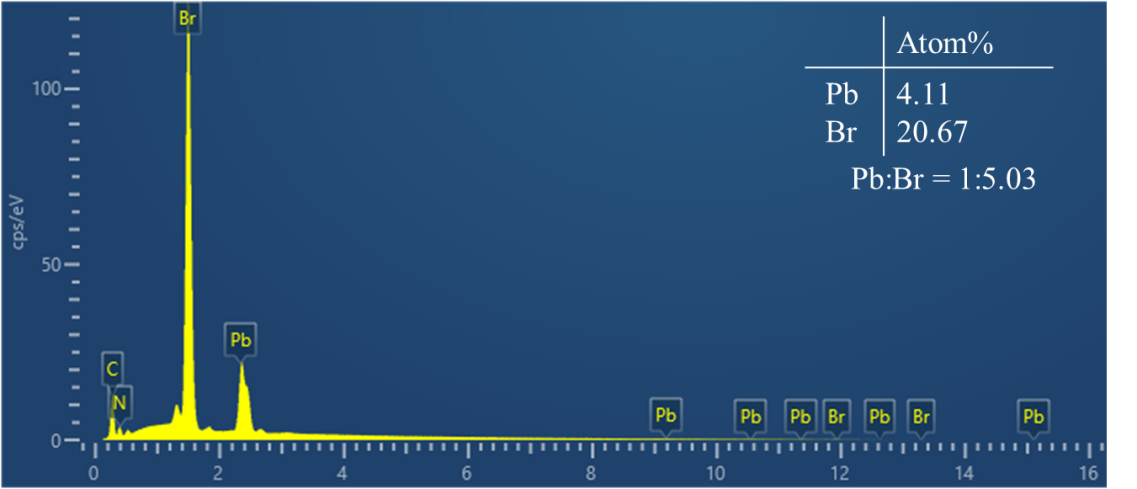
 (b)

**Figure S6**. The SEM image and elemental mapping images of Pb, Br, C and N elements (a), EDX analysis result (b) of G-[DADPA]PbBr_5_.


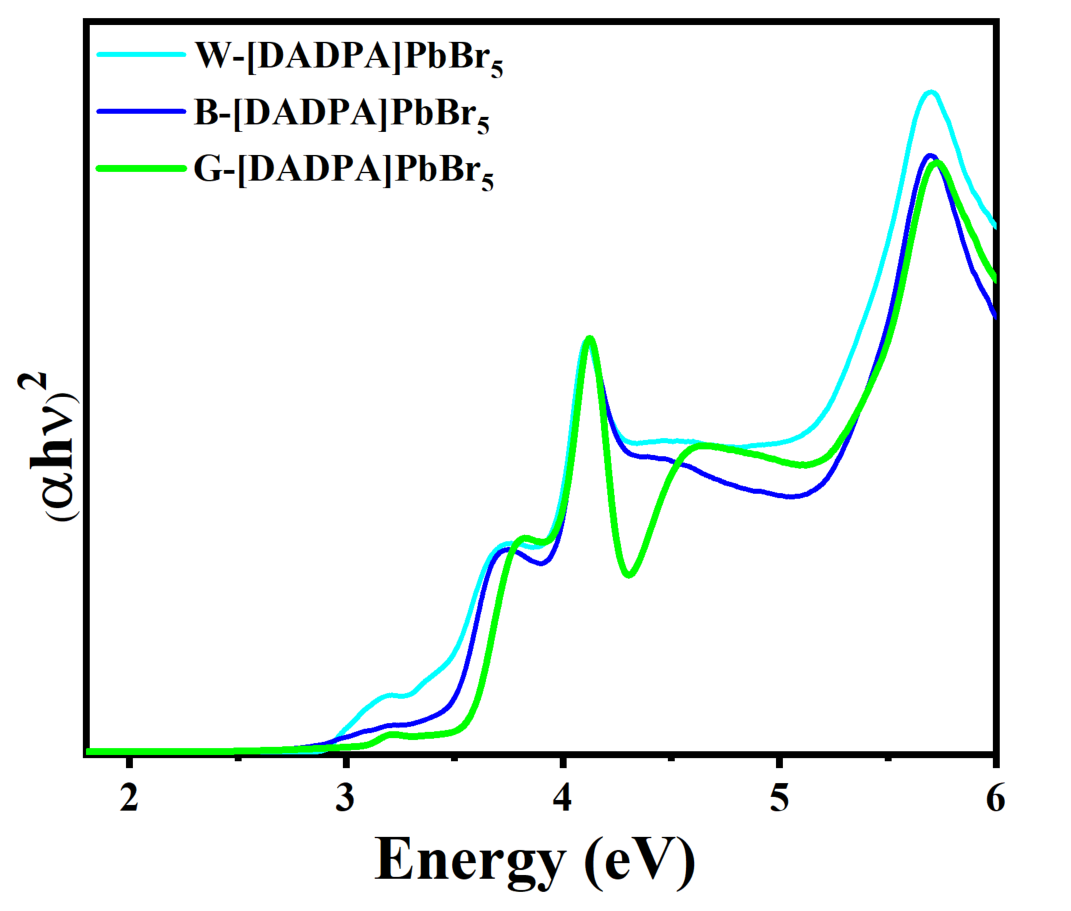


**Figure S7.** Tauc plots of W-, B- and G-[DADPA]PbBr_5_ fitted by direct bandgap model, respectively.

(a)

(b)

**Figure S8.** The emission wavelength dependent PL excitation spectra (a) and excitation wavelength dependent PL emission spectra (b) of W-[DADPA]PbBr_5_.

(a)

(b)

**Figure S9.** The emission wavelength dependent PL excitation spectra (a) and excitation wavelength dependent PL emission spectra (b) of B-[DADPA]PbBr_5_.

(a)

(b)

(c)

**Figure S10.** The emission wavelength dependent PL excitation spectra (a) and excitation wavelength dependent PL emission spectra (b) of G-[DADPA]PbBr_5_.

(a)

(b)

(c)

**Figure S11.** 3D PL excitation and emission correlation maps of W-[DADPA]PbBr_5_ (a); B-[DADPA]PbBr_5_ (a) and G-[DADPA]PbBr_5_ (c).


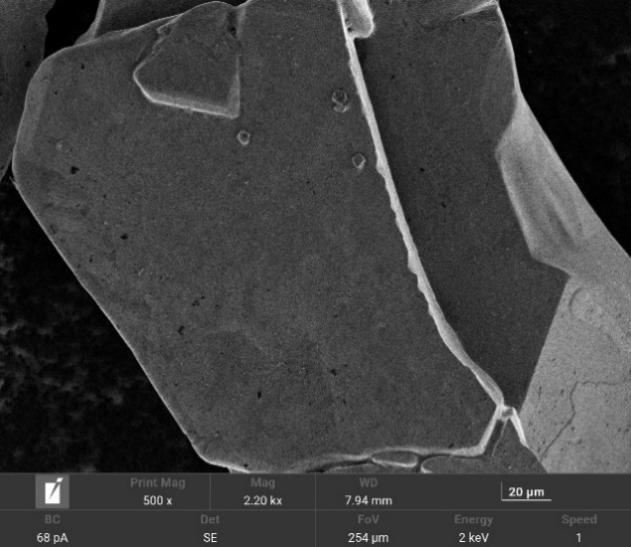

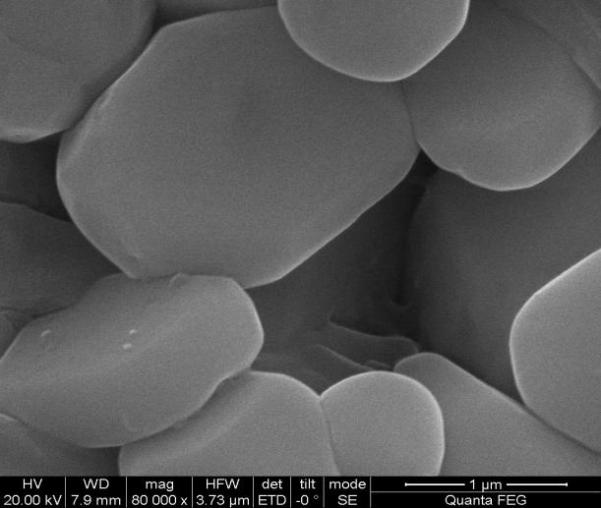
(a)


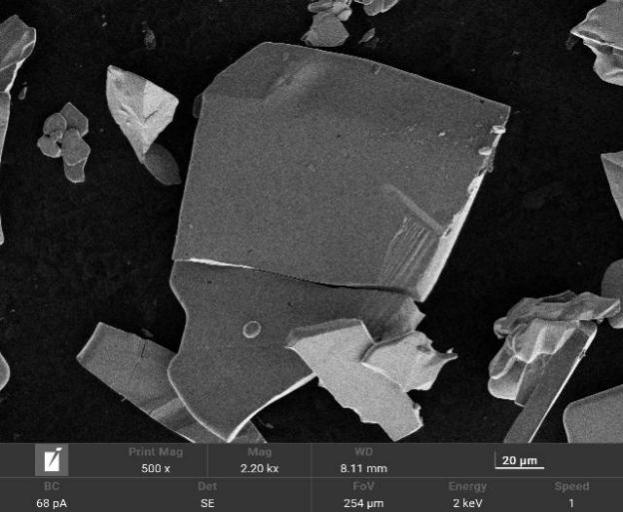

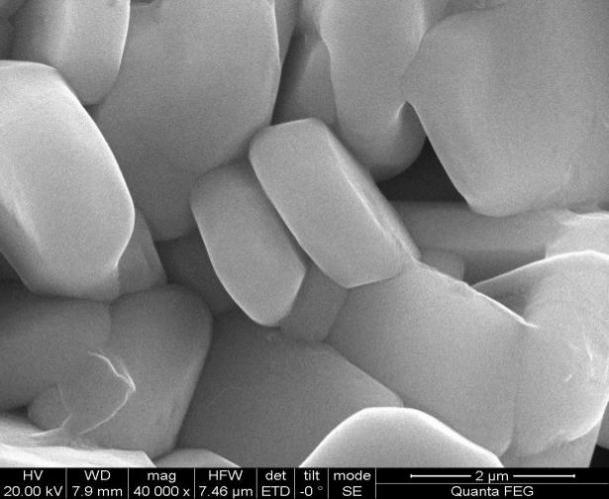
(b)


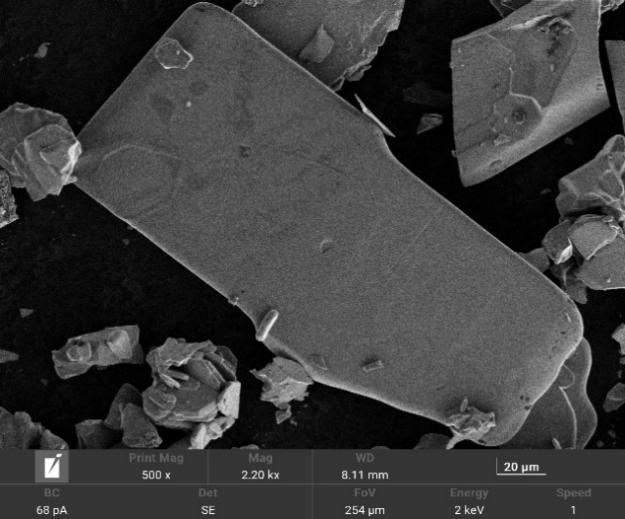

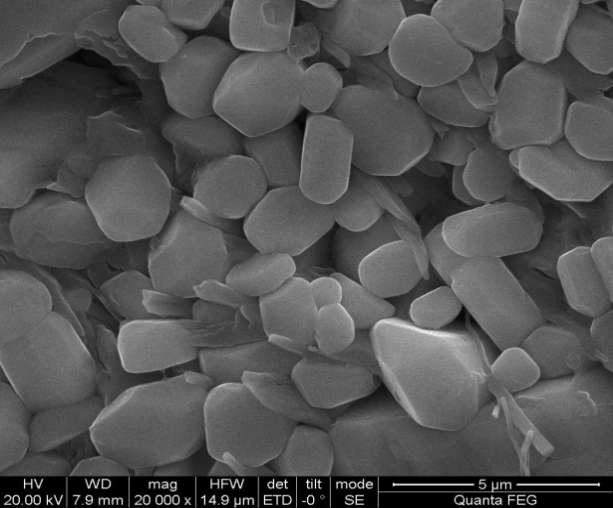
(c)

**Figure S12.** The SEM photos of bulk crystals and microscale crystals of W-[DADPA]PbBr_5_ (a), B-[DADPA]PbBr_5_ (b) and G-[DADPA]PbBr_5_ (c).

(a)

(b)

(c)

**Figure S13.** Comparison of the PL emission spectra of bulk and microscale crystals for W-[DADPA]PbBr_5_ (a), B-[DADPA]PbBr_5_ (b) and G-[DADPA]PbBr_5_ (c).

**Figure S14.** PL decay curve of W-[DADPA]PbBr_5_ monitoring at 488 nm at room temperature.

(a)

(b)


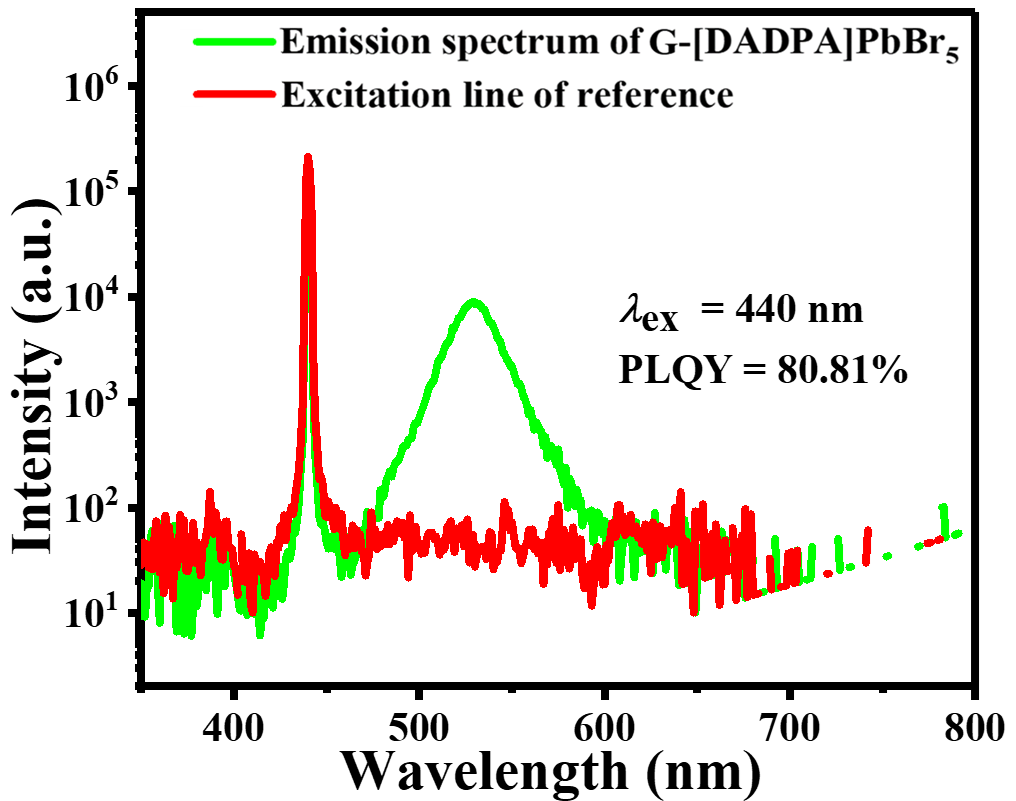
 (c)

**Figure S15.** PLQYs of W-[DADPA]PbBr_5_ (a), B-[DADPA]PbBr_5_ (b) and G-[DADPA]PbBr_5_ (c) at room temperature.


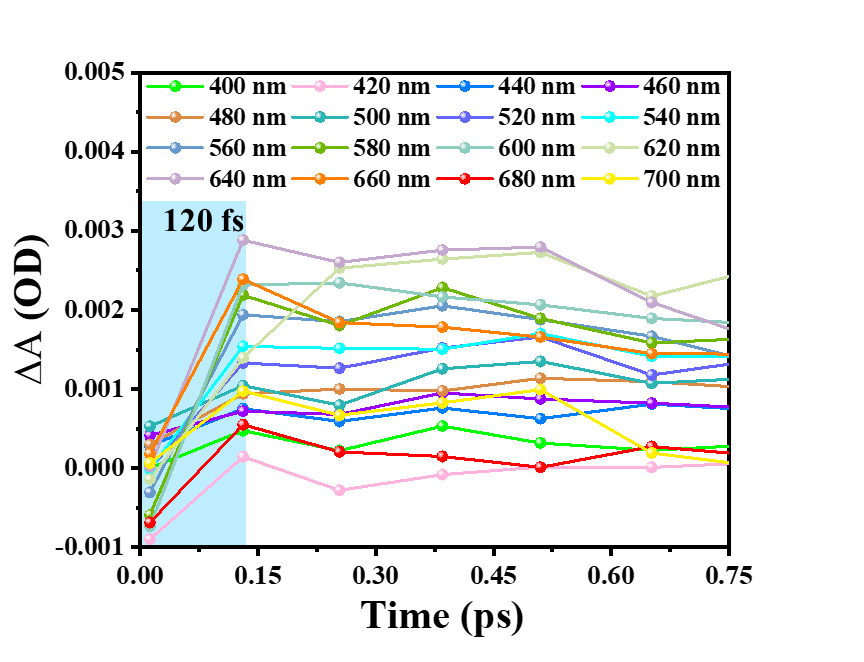
(a)


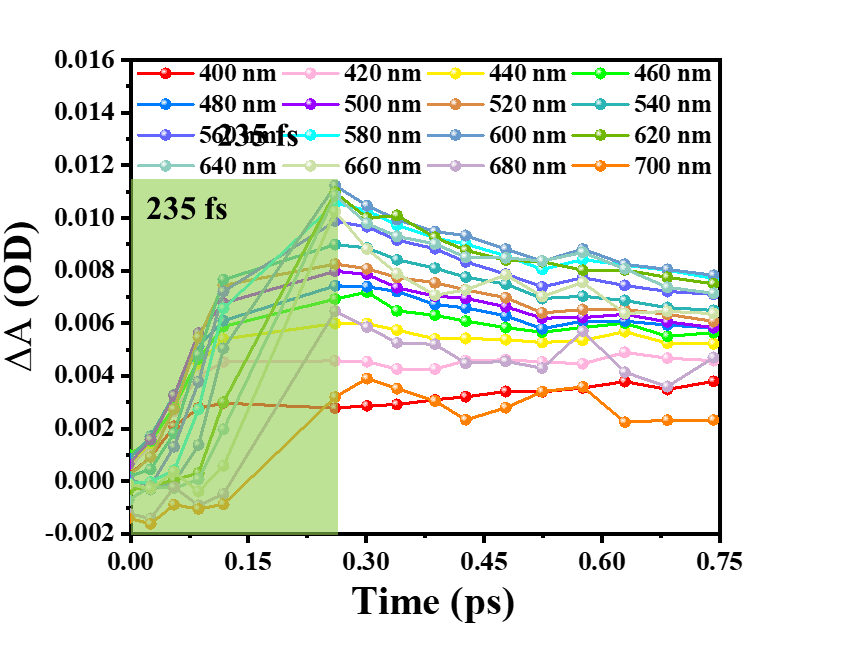
 (b)

**Figure S16.** PIA onsets of W-[DADPA]PbBr_5_ (a) and G-[DADPA]PbBr_5_ (b) probed at different wavelengths.

(a)

(b)

(c)

**Figure S17.** Temperature dependent PL emission spectra of W-[DADPA]PbBr_5_ (a), B-[DADPA]PbBr_5_ (b) and G-[DADPA]PbBr_5_ (c).

(a) (b)

(c)

**Figure S18.** Temperature dependent emission wavelength (red) with the contribution of thermal expansion (green), and electronic-phonon interactions (blue) of W-[DADPA]PbBr_5_ (a), B-[DADPA]PbBr_5_ (b) and G-[DADPA]PbBr_5_ (c). The temperature-dependent emission peak shift is related to the synergistic effects of thermal expansion (TE) and electron-phonon (EP) interaction. Using the one-oscillator model and assuming a linear relationship between thermal expansion and temperature, the evolution of emission energy with temperature *E*_g_(*T*) can be fitted according to the follow model:

$$E_{g}\left( T \right)=E_{0}+A_{\mathrm{TE}}T+A_{\mathrm{EP}}\left[ \frac{2}{\exp\left( \frac{h\omega T}{k_{B}} \right)-1}+1 \right]$$

where *E*_0_ is the unrenormalized band gap, *A*_TE_ and *A*_EP_ are the weight of TE and EP interactions, respectively, *hω* is the average optical phonon energy and *k*_B_ is Boltzmann’s constant.


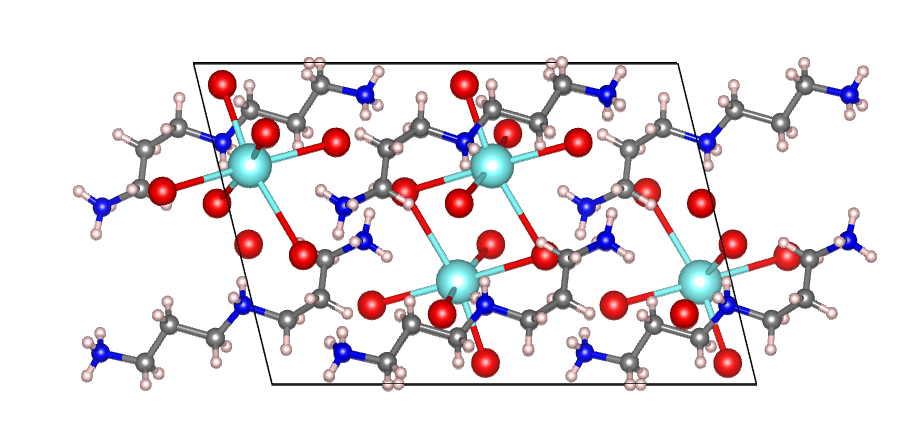


**Figure S19.** The structural model of G-[DADPA]PbBr_5_ in the theoretical calculation.


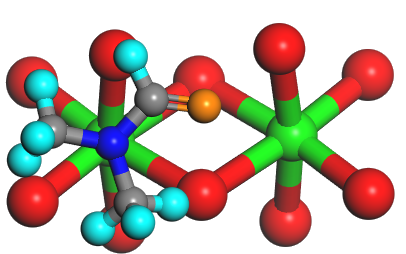

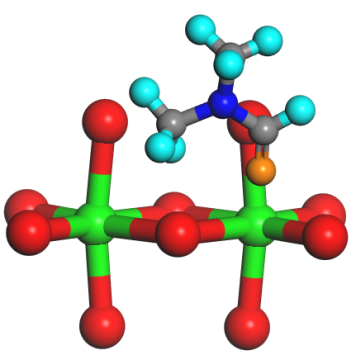

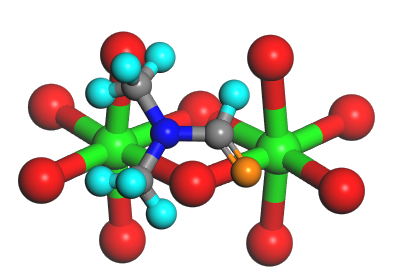
(a)


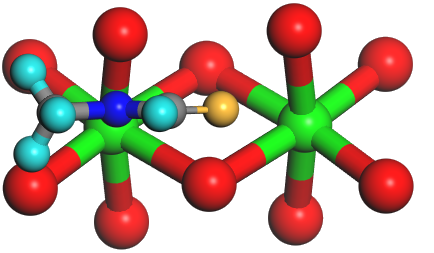

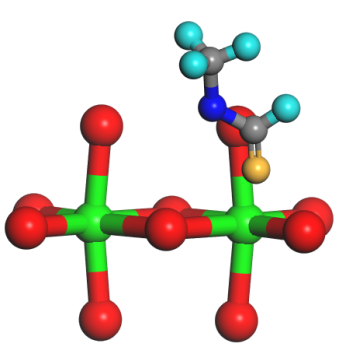

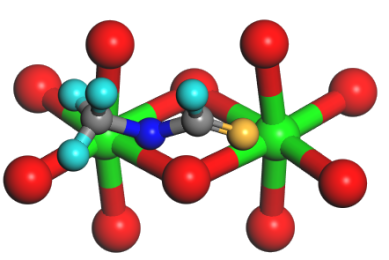
(b)

**Figure S20**. The adsorption models of DMA (a) and NMA (b) molecule on the surface of simplified Pb_2_Br_10_ dimers via N-, O- and double N-, O-band connections. Pb green, Br red, N blue, O yellow, C grey, H light blue.


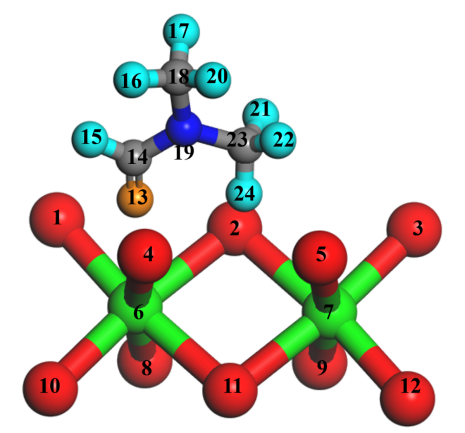
(a)
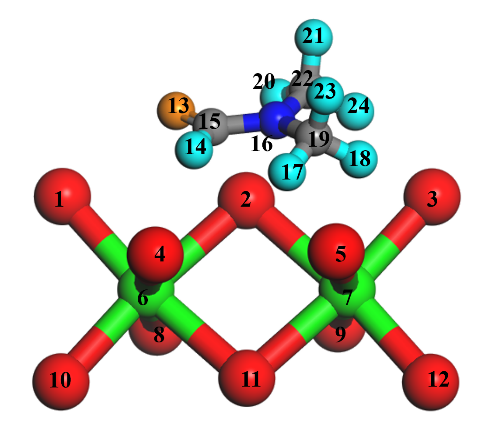
(b)

| **D-H···A** | **d(D-H)** | **d(H···A)** | **d(D···A)** |
| --- | --- | --- | --- |
| O-band connection | | | |
| C14-H15---Br4 | 1.115Å | 2.836Å | 2.318Å |
| Double N, O-bond connection | | | |
| C15-H14---Br4 | 1.115Å | 2.410Å | 3.286Å |
| C15-H14---Br2 | 1.115Å | 3.033Å | 2.375Å |
| C19-H17---Br4 | 1.099Å | 3.285Å | 4.155Å |
| C19-H17---Br2 | 1.099Å | 3.591Å | 3.435Å |
| C19-H17---Br5 | 1.099Å | 1.988Å | 2.468Å |
| C22-H20---Br2 | 1.097Å | 2.296Å | 2.713Å |

**Figure S21**. The adsorption models (a and b) and corresponding hydrogen bonding interactions (c) of DMA molecule on the [DADPA]PbBr_5_ perovskite surface via O- (a) and double N-, O-band connections (b).


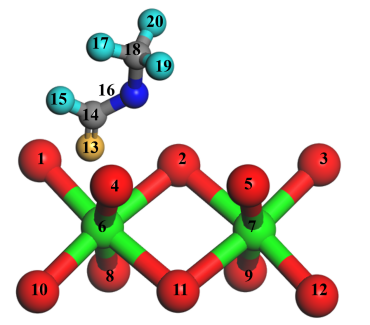
(a)
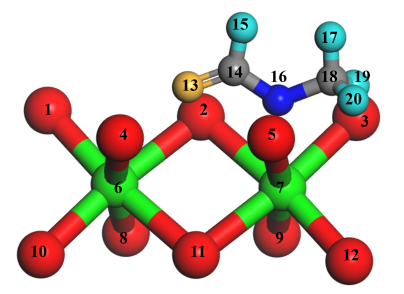
(b)
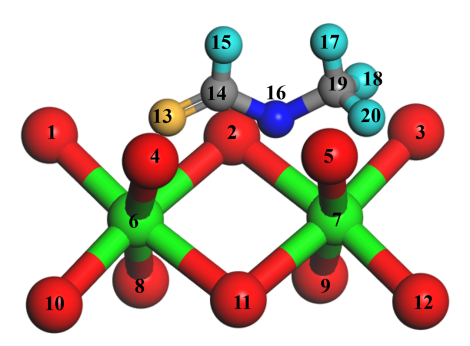
(c)

| **D-H···A** | **d(D-H)** | **d(H···A)** | **d(D···A)** |
| --- | --- | --- | --- |
| O-band connection | | | |
| C14-H15···Br1 | 1.112 | 3.505 | 3.184 |
| C14-H15···Br4 | 1.112 | 2.792 | 2.199 |
| N-band connection | | | |
| C14-H15···Br2 | 1.112 | 3.332 | 2.472 |
| C14-H15···Br5 | 1.112 | 3.088 | 2.368 |
| C18-H20···Br5 | 1.099 | 2.324 | 2.489 |
| C18-H20···Br3 | 1.099 | 2.763 | 2.221 |
| C18-H19···Br3 | 1.100 | 1.262 | 2.221 |
| C18-H17···Br3 | 1.099 | 3.011 | 2.221 |
| C18-H17···Br5 | 1.099 | 3.144 | 2.489 |
| Double N, O-bond connection | | | |
| C14-H15---Br4 | 1.112 | 3.203 | 2.741 |
| C14-H15---Br2 | 1.112 | 2.828 | 1.859 |
| C19-H20---Br5 | 1.101 | 1.540 | 2.225 |
| C19-H20---Br3 | 1.101 | 2.592 | 2.562 |
| C19-H18---Br2 | 1.100 | 3.007 | 2.854 |
| C19-H18---Br3 | 1.100 | 1.750 | 2.562 |
| C19-H17---Br2 | 1.009 | 3.473 | 2.854 |
| C19-H17---Br3 | 1.009 | 3.419 | 2.562 |
| C19-H17---Br5 | 1.009 | 2.706 | 2.225 |

**Figure S22**. The adsorption models (a-c) and corresponding hydrogen bonding interactions (c) of DMA molecule on the [DADPA]PbBr_5_ perovskite surface via O- (a), N- (b) and double N-, O-band (c) connections.

(a)(b) (c)(d)

**Figure S23.** The experimental powder X-ray diffraction (PXRD) patterns (a, c) and PL emission spectra (b, d) of W-[DADPA]PbBr_5_ after soaking in various organic solvents (ACN = acetonitrile, DCM = dichloromethane, EAC = acetylacetone, NMF= N-Methylacetamide, DMF= N,N-Dimethylformamide, DMA= N,N-Dimethylacetamide, CP = acetone, THF = tetrahydrofuran).

(a)(b) (c)(d)

**Figure S24.** The experimental powder X-ray diffraction (PXRD) patterns (a, c) and PL emission spectra (b, d) of B-[DADPA]PbBr_5_ after soaking in various organic solvents (ACN = acetonitrile, DCM = dichloromethane, EAC = acetylacetone, NMF= N-Methylacetamide, DMF= N,N-Dimethylformamide, DMA= N,N-Dimethylacetamide, CP = acetone, THF = tetrahydrofuran).


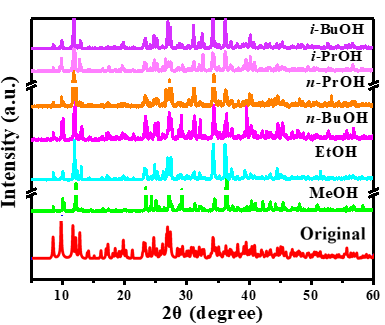
(a)(b)


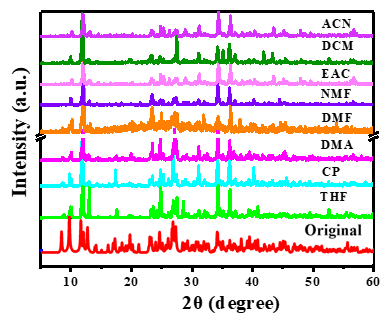
(c)(d)

**Figure S25.** The experimental powder X-ray diffraction (PXRD) patterns (a, c) and PL emission spectra (b, d) of G-[DADPA]PbBr_5_ after soaking in various organic solvents. (ACN = acetonitrile, DCM = dichloromethane, EAC = acetylacetone, NMF= N-Methylacetamide, DMF= N,N-Dimethylformamide, DMA= N,N-Dimethylacetamide, CP = acetone, THF = tetrahydrofuran).

(a)

(b)

**Figure S26.** Comparisons of experimental PXRD patterns (a) and emission spectra (b) of W-[DADPA]PbBr_5_ after storing in humid air for one month.

(a)

(b)

**Figure S27.** Comparisons of experimental PXRD patterns (a) and emission spectra (b) of B-[DADPA]PbBr_5_ after storing in humid air for one month.

(a)

(b)

**Figure S28.** Comparisons of experimental PXRD patterns (a) and emission spectra (b) of G-[DADPA]PbBr_5_ after storing in humid air for one month.

(a)

(b)

**Figure S29.** Comparisons of experimental PXRD patterns (a) and PL emission spectra (b) of W-[DADPA]PbBr_5_ after exposure under strong UV light irradiation (300 W/cm^2^, Xe lamp) for 24h.

(a)

(b)

**Figure S30.** Comparisons of experimental PXRD patterns (a) and PL emission spectra (b) of B-[DADPA]PbBr_5_ after exposure under strong UV light irradiation (300 W/cm^2^, Xe lamp) for 24h.

(a)

(b)

**Figure S31.** Comparisons of experimental PXRD patterns (a) and PL emission spectra (b) of G-[DADPA]PbBr_5_ after exposure under strong UV light irradiation (300 W/cm^2^, Xe lamp) for 24h.

**Figure S32.** The thermogravimetric analysis (TGA) curves of W-[DADPA]PbBr_5_, B-[DADPA]PbBr_5_ and G-[DADPA]PbBr_5_.

(a)

(b)

**Figure S33.** Experimental PXRD patterns (a) and PL emission spectra (b) of W-[DADPA]PbBr_5_ after constant heating at different temperature from 80 °C to 160 °C over 30 minutes.

(a)

(b)

**Figure S34.** Experimental PXRD pattern (a) and PL emission spectra (b) of B-[DADPA]PbBr_5_ after constant heating at different temperature from 80 °C to 160 °C over 30 minutes.

(a)

(b)

**Figure S35.** Experimental PXRD pattern (a) and PL emission spectra (b) of G-[DADPA]PbBr_5_ after constant heating at different temperature from 80 °C to 160 °C over 30 minutes.

**
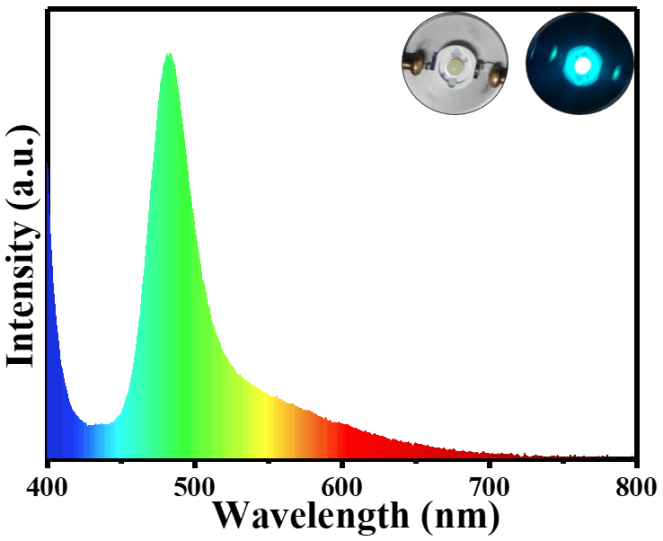
**(a)

(b)

(c)

**Figure S36.** (a) Electroluminescence (EL) emission spectrum, (b) Drive current-dependent EL emission spectra, (c) Luminous efficiency of fabricated blue LED based on B-[DADPA]PbBr_5_ on a UV chip.


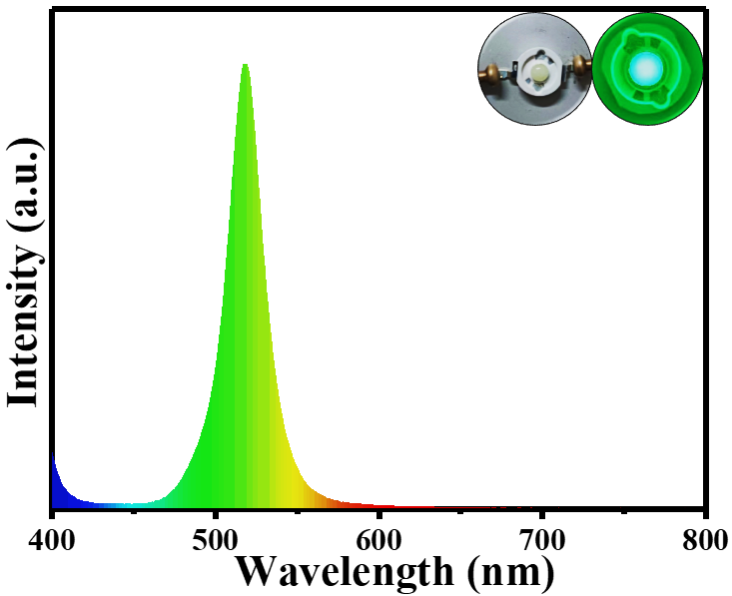
 (a)

(b)

(c)

**Figure S37.** (a) Electroluminescence (EL) emission spectrum, (b) Drive current-dependent EL emission spectra, (c) Luminous efficiency of fabricated green LED based on G-[DADPA]PbBr_5_ on a UV chip.

(a)

(b)

(c)

**Figure S38.** (a) Electroluminescence (EL) emission spectrum, (b) Drive current-dependent EL emission spectra, (c) Luminous efficiency of fabricated white LED based on W-[DADPA]PbBr_5_ on a UV chip.

(a)

(b)
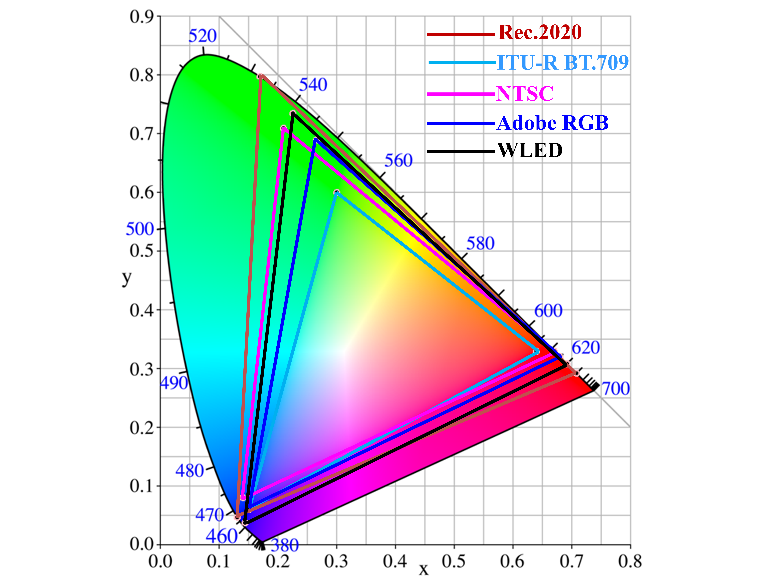
 (c)

**Figure S39.** (a) Summary of the chromaticity coordinates of G-[DADPA]PbBr_5_ and reported green light emitting metal halides, as well as ITU-R BT.709, NTSC, the Rec. 2020 standards and ITU-R BT.709; (b) The EL spectra of LED fabricated by green G-[DADPA]PbBr_5_ phosphor and commercial red phosphor K_2_SiF_6_:Mn^4+^ on a 455 nm blue LED chip at 20 mA drive current (c) Color gamut of the fabricated LED (black line), and color space of the Rec.2020 (dark red line), ITU-R BT.709 (light blue line), NTSC standard (purple line) and Adobe RGB (blue line) in the CIE-1931 diagram.


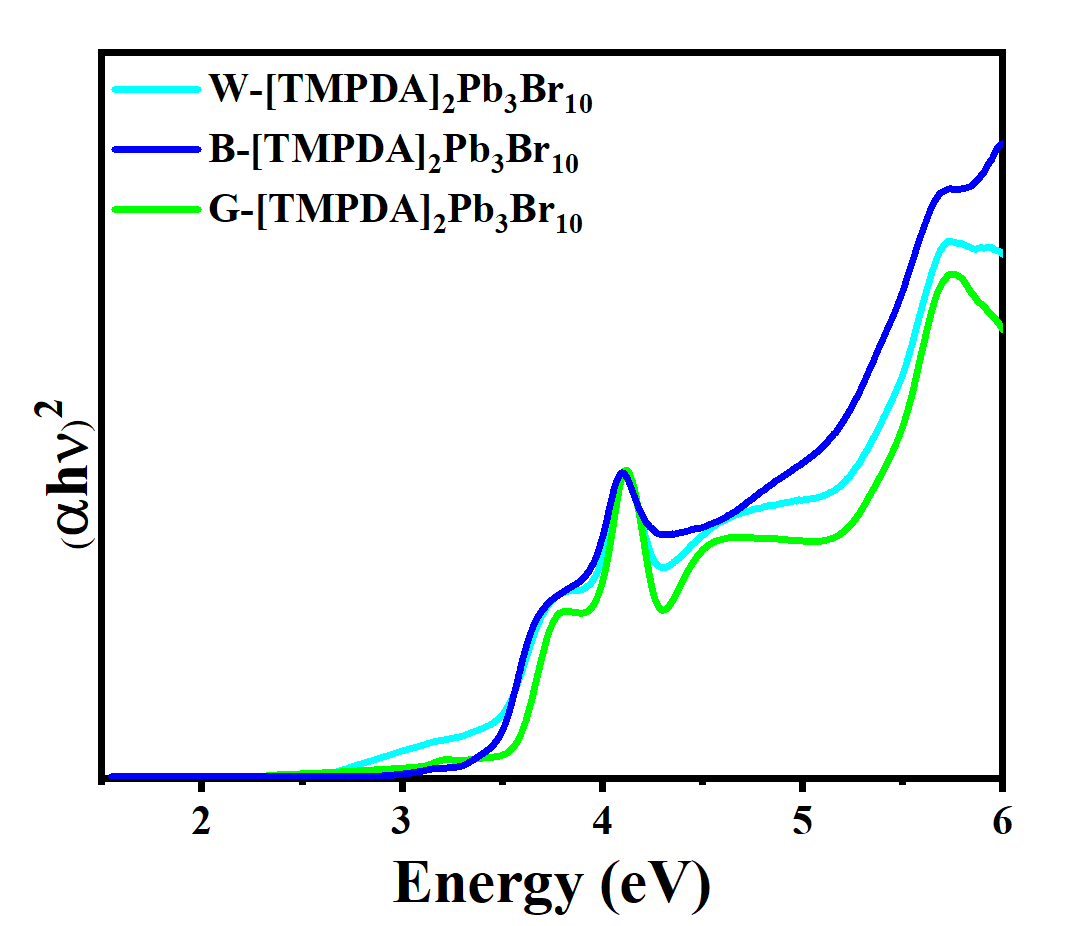


**Figure S40.** Tauc plots of W-, B- and G-[TMPDA]_2_Pb_3_Br_10_ fitted by direct bandgap, respectively.


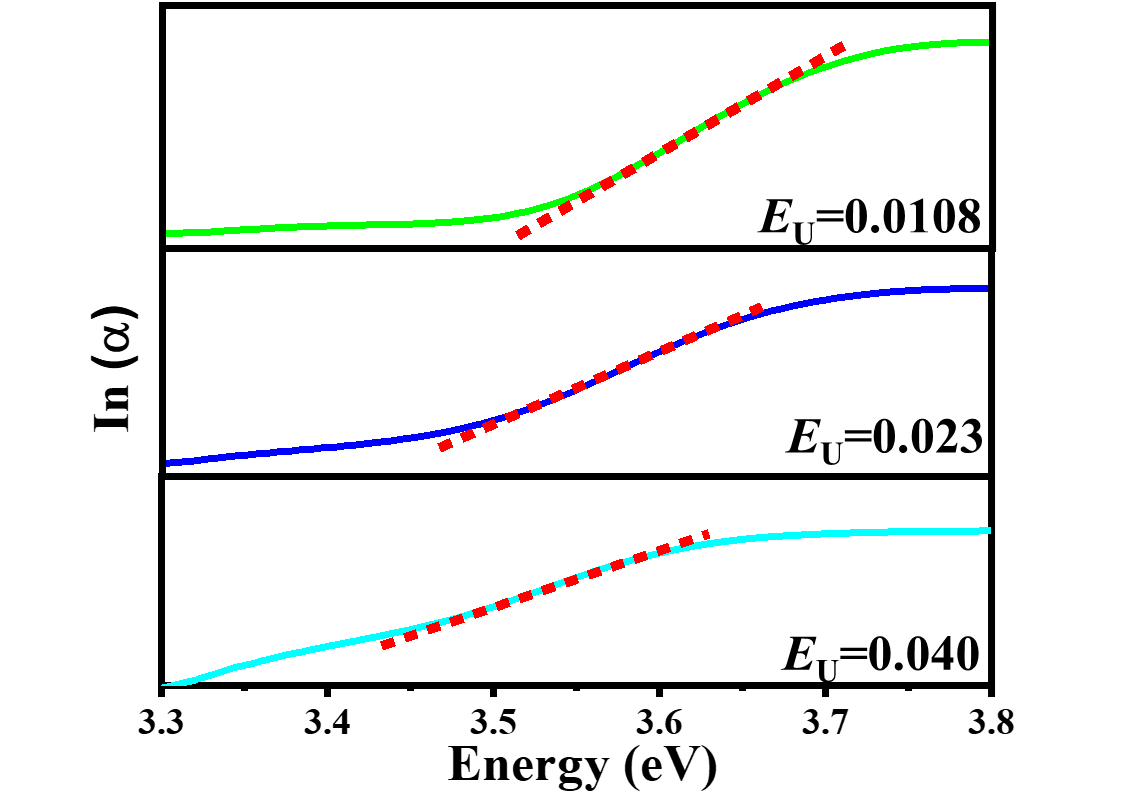


**Figure S41.** Logarithmic absorption coefficient extracted from optical absorption spectra as a function of photon energy, and corresponding Urbach energies for W-[TMPDA]_2_Pb_3_Br_10_, B-[TMPDA]_2_Pb_3_Br_10_ and G-[TMPDA]_2_Pb_3_Br_10_.


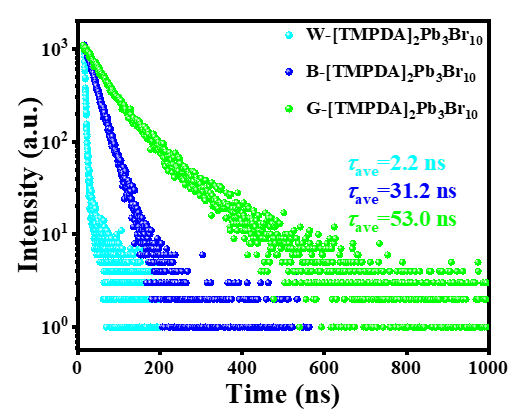


**Figure S42.** Time-resolved PL spectroscopy of W-[TMPDA]_2_Pb_3_Br_10_, B-[TMPDA]_2_Pb_3_Br_10_ and G-[TMPDA]_2_Pb_3_Br_10_.

(a)

(b)

(c)

**Figure S43.** PLQYs of W-[TMPDA]_2_Pb_3_Br_10_ (a), B-[TMPDA]_2_Pb_3_Br_10_ (b) and G-[TMPDA]_2_Pb_3_Br_10_ (c) at room temperature.

**Table S1**. Summary of the photoluminescence parameters of some representative green light emitting metal halides.

| **Material** | **Emission (nm)** | **CIE** | **PLQY** | **FWHM (nm)** | **Lifetime** | **Reference** |
| --- | --- | --- | --- | --- | --- | --- |
| **Single Crystalline Pb-based Halides** | | | | | | |
| **G-[DADPA]PbBr_5_** | **535** | **(0.23, 0.73)** | **80.80 %** | **27** | **57.7 ns** | **This work** |
| **[TMPDA]Pb_3_Br_10_** | **519** | **(0.12, 0.70)** | **71.59 %** | **27** | **53.03 ns** | **This work** |
| C_4_N_2_H_14_PbCl_4_ | 545 | (0.29,0.39) | 6 | 179 | 20 ns | 6 |
| [bmpy]_6_Pb_3_Br_12_ | 522 | (0.26, 0.39) | 12 % | 134 | 36.78 ns | 7 |
| [bmpy]_9_[MnBr_4_]_2_[Pb_3_Br_11_] | 528 | (0.29, 0.65) | 49.8 % | 67 | 114.3 μs | 8 |
| [bmpy]_9_[ZnBr_4_]_2_[Pb_3_Br_11_] | 564 | (0.40,0.53) | 7 % | 68 | 36 ns | 9 |
| [bmpy]_9_[ZnCl_4_]_2_[Pb_3_Cl_11_] | 512 | (0.16, 0.55) | 100 % | 61 | 0.54 μs | 10 |
| (EDBE)[PbI_4_] | 515 | (0.29, 0.58) | 0.5% | 70 | -- | 11 |
| **Perovskite Quantum Dots and Nanocrystals** | | | | | | |
| CsPbBr_3_ | 507 | (0.23, 0.63) | 64.56 % | 23 | 6.6 ns | 12 |
| MAPbBr_3_ | 527 | -- | 92 % | 19 | 35 ns | 13 |
| FAPbBr_3_ NC | 530 | -- | 85 % | 22 | 25 ns | 14 |
| FAPbBr_3_ film | 530 | (0.17, 0.77) | 92% | 22 | 24.2 ns | 15 |
| CsPb_2_Br_5_ | 520 | -- | 85 % | 22 | 32 ns | 16 |
| Cs_4_PbBr_6_ | 514 | -- | 46 % | 19 | 36.4 ns | 17 |
| Cs_3_Cu_2_Cl_3_ | 515 | (0.19, 0.42) | 91.3 % | 91 | 112.4 μs | 18 |
| Cs_3_MnBr_5_ | 520 | -- | 49 % | 42 | 0.29 ms | 19 |
| Cs_3_MnI_5_ | 540 | -- | 73 % | 48 | 25.6 μs | 20 |
| **Manganese halide materials** | | | | | | |
| [bmpy]_2_MnBr_4_ | 528 nm | (0.279,0.645) | 81.08 % | -- | 325.57 μs | 21 |
| [P14]MnBr_4_ | 520 | (0.23, 0.68) | 81% | 55 | 358 μs | 22 |
| [PP14]MnBr_4_ | 527 | (0.28, 0.66) | 55% | 64 | 361 μs |
| (TMS)_2_MnBr_4_ | 517 | (0.18, 0.70) | 69.8 % | 42 | 306.81 μs | 23 |
| [PPh4]_2_MnBr_4_ | 522 | -- | 98 % | -- | 317 μs | 24 |
| (C_38_H_34_P_2_)MnBr_4_ | 217 | -- | 95 % | 51 | 318μs | 25 |
| (diisopropylammonium)_2_MnBr_4_ | 525 | -- | 62.2 % | 62.9 | 1.44 ns | 26 |
| (C_4_NOH_10_)_2_MnCl_4_ | 520 | (0.24,0.64) | 39 % | -- | 3.36 ms | 27 |
| (C_5_H_6_N)_2_MnBr_4_ | 521 | -- | 95 % | 42 | 261μs | 28 |
| (Bz(Me)_3_N)_2_MnCl_4_ | 547 | -- | 78 % | 71.8 | 3.79 ms | 29 |

bmpy = 1-butyl-1-methylpyrrolidinium; EDBE = 2,2’-(ethylenedioxy)bis(ethylammonium); P14 = N-butyl-N-methylpyrrolidinium; PP14 = N-butyl-N-methylpiperidinium; TMS = trimethylsulfonium; PPh_4_ = Tetraphenylphosphonium; BzMe_3_NCl = benzyl-N,N,N-trimethylammonium chloride.

**Table S2**. Average ratio of Pb ang Br based on the ICP-MS and EDS results (atom %)

|  | Pb : Br (At%) |
| --- | --- |
| W-[TMPDA]_2_Pb_3_Br_10_ | 3:9.24 |
| B-[TMPDA]_2_Pb_3_Br_10_ | 3:9.71 |
| G-[TMPDA]_2_Pb_3_Br_10_ | 3:10.16 |

**Table S3**. The summary of photophysical properties for W-, B- to G-[TMPDA]_2_Pb_3_Br_10_.

|  | *λ*_em_  (nm) | FWHM  (nm) | PLQY  (%) | Lifetime  (ns) | *K*_r_ (×10^7^)  (s^-1^) | *K_n_*_r_ (×10^7^)  (s^-1^) |
| --- | --- | --- | --- | --- | --- | --- |
| W-[TMPDA]_2_Pb_3_Br_10_ | 445 | 90 | 1 | 2.2 | 0.45 | 45.42 |
| B-[TMPDA]_2_Pb_3_Br_10_ | 489 | 30 | 37.9 | 31.2 | 1.21 | 2.05 |
| G-[TMPDA]_2_Pb_3_Br_10_ | 535 | 25 | 71.6 | 53 | 1.35 | 0.72 |

**Table S4**. Crystal data and structural refinements for compounds W-[DADPA]PbBr_5_, B-[DADPA]PbBr_5_ and G-[DADPA]PbBr_5_.

| Compound | W-[DADPA]PbBr_5_ | B-[DADPA]PbBr_5_ | G-[DADPA]PbBr_5_ |
| --- | --- | --- | --- |
| chemical formula | C_6_H_20_N_3_PbBr_5_ | C_6_H_20_N_3_PbBr_5_ | C_6_H_20_N_3_PbBr_5_ |
| fw | 740.95 | 740.99 | 740.95 |
| Space group | *P*2_1_*/c* (No.14) | *P*2_1_*/c* (No.14) | *P*2_1_*/c* (No.14) |
| *a*/Å | 10.484(3) | 10.4777(3) | 10.4829(19) |
| *b/*Å | 11.036(3) | 11.0286(3) | 11.038(2) |
| *c*/Å | 15.372(4) | 15.3502(5) | 15.373(3) |
| *β/º* | 103.796(5) | 103.7810(10) | 103.763(2) |
| *V*(Å^3^) | 1727.3(8) | 1722.72(9) | 1727.7(6) |
| Z | 4 | 4 | 4 |
| *D*_calcd_ (g∙cm^-3^) | 2.849 | 2.857 | 2.849 |
| Temp (*K*) | 296(2) | 296(2) | 296(2) |
| *μ* (mm^-1^) | 21.315 | 21.371 | 21.309 |
| *F* (000) | 1336 | 1336 | 1336 |
| Reflections collected | 10760 | 21348 | 20187 |
| Unique reflections | 3838 | 3046 | 3975 |
| GOF on *F* ^2^ | 1.059 | 1.053 | 1.024 |
| *R*_1_,*wR*_2_ (*I* > 2*σ*(*I*))^a^ | 0.0554/ 0.1004 | 0.0437/ 0.1330 | 0.0431/ 0.0753 |
| *R*_1_,*wR*_2_ (all data) | 0.0981/ 0.1147 | 0.0512/ 0.1395 | 0.0866/ 0.0864 |

^a^$R_{1}=\Sigma\left|\left|F_{0}\right|-\left|F_{c}\right|\right|/\Sigma\left|F_{0}\right|$,$wR_{2}=[\Sigma(F_{_{0}}^{2}-F_{c}^{2})/\Sigma w(F_{0})^{2}]^{1/2}$

**Table S5**. Selected bond lengths (Å) for compounds W-[DADPA]PbBr_5_, B-[DADPA]PbBr_5_ and G-[DADPA]PbBr_5_.

|  | W-[DADPA]PbBr_5_ | B-[DADPA]PbBr_5_ | G-[DADPA]PbBr_5_ |
| --- | --- | --- | --- |
| Pb(1)-Br(4) | 2.8388(14) | 2.8383(11) | 2.8419(10) |
| Pb(1)-Br(3) | 2.9429(17) | 2.9385(11) | 2.9438(12) |
| Pb(1)-Br(5) | 3.0102(15) | 3.0077(11) | 3.0117(11) |
| Pb(1)-Br(2) | 3.0472(15) | 3.0434(11) | 3.0493(11) |
| Pb(1)-Br(1) | 3.1620(16) | 3.1565(11) | 3.1622(12) |
| Pb(1)-Br(1)#1 | 3.4469(15) | 3.4402(11) | 3.4432(11) |

Symmetry transformations used to generate equivalent atoms: #1 -*x*+1, -*y*+1, -*z*+1

**Table S6**. Selected bond angles (°) for compounds W-[DADPA]PbBr_5_, B-[DADPA]PbBr_5_ and G-[DADPA]PbBr_5_.

| Br(3)-Pb(1)-Br(2)  Br(5)-Pb(1)-Br(2)  Br(4)-Pb(1)-Br(1)  Br(3)-Pb(1)-Br(1)  Br(5)-Pb(1)-Br(1)  Br(2)-Pb(1)-Br(1)  Br(4)-Pb(1)-Br(1)#1  Br(3)-Pb(1)-Br(1)#1  Br(5)-Pb(1)-Br(1)#1  Br(2)-Pb(1)-Br(1)#1  Br(1)-Pb(1)-Br(1)#1 | W-[DADPA]PbBr_5_ | B-[DADPA]PbBr_5_ | G-[DADPA]PbBr_5_ |
| --- | --- | --- | --- |
| Br(4)-Pb(1)-Br(3) | 88.78(3) | 88.76(3) | 88.71(4) |
| Br(4)-Pb(1)-Br(5) | 90.00(3) | 89.94(3) | 90.07(4) |
| Br(3)-Pb(1)-Br(5) | 100.98(3) | 100.86(3) | 100.99(4) |
| Br(4)-Pb(1)-Br(2) | 91.06(3) | 91.19(3) | 91.10(4) |
| Br(3)-Pb(1)-Br(2) | 90.81(3) | 90.80(3) | 90.81(4) |
| Br(5)-Pb(1)-Br(2) | 168.18(3) | 168.31(4) | 168.16(4) |
| Br(4)-Pb(1)-Br(1) | 94.85(3) | 94.92(3) | 94.92(4) |
| Br(3)-Pb(1)-Br(1) | 171.98(3) | 171.99(3) | 172.01(4) |
| Br(5)-Pb(1)-Br(1) | 86.19(3) | 86.29(3) | 86.14(4) |
| Br(2)-Pb(1)-Br(1) | 81.99(3) | 82.02(3) | 82.02(4) |

**Table S7.** Hydrogen bonds data for compound W-[DADPA]PbBr_5_.

| D-H···A | d(D-H) | d(H···A) | d(D···A) | <(DHA) |
| --- | --- | --- | --- | --- |
| N(1)-H(1C)∙∙∙Br(2) | 0.89 | 2.63 | 3.3723 | 141 |
| N(1)-H(1D)∙∙∙Br(3) | 0.89 | 2.58 | 3.3741 | 148 |
| N(1)-H(1E)∙∙∙Br(1) | 0.89 | 2.90 | 3.4460 | 121 |
| N(2)-H(2C)∙∙∙Br(1) | 0.89 | 2.78 | 3.3364 | 122 |
| N(2)-H(2C)∙∙∙Br(3) | 0.89 | 2.85 | 3.4419 | 125 |
| N(2)-H(2D)∙∙∙Br(2) | 0.89 | 2.61 | 3.4841 | 166 |
| N(2)-H(2E)∙∙∙Br(1) | 0.89 | 2.64 | 3.4832 | 158 |
| N(3)-H(3C)∙∙∙Br(5) | 0.89 | 2.46 | 3.2934 | 155 |
| C(3)-H(3D)∙∙∙Br(2) | 0.89 | 2.50 | 3.3117 | 152 |
| C(6)-H(6B)∙∙∙Br(1) | 0.97 | 2.87 | 3.7357 | 149 |

**Table S8.** Hydrogen bonds data for compound B-[DADPA]PbBr_5_.

| D-H···A | d(D-H) | d(H···A) | d(D···A) | <(DHA) |
| --- | --- | --- | --- | --- |
| N(1)-H(1A)∙∙∙Br(1) | 0.89 | 2.90 | 3.465 | 123 |
| N(1)-H(1C)∙∙∙Br(4) | 0.89 | 2.60 | 3.354 | 143 |
| N(1)-H(1D)∙∙∙Br(1) | 0.89 | 2.58 | 3.372 | 149 |
| N(1)-H(1E)∙∙∙Br(3) | 0.89 | 2.67 | 3.485 | 152 |
| N(2)-H(2C)∙∙∙Br(1) | 0.89 | 2.63 | 3.332 | 136 |
| N(2)-H(2C)∙∙∙Br(3) | 0.89 | 2.62 | 3.475 | 161 |
| N(2)-H(2D)∙∙∙Br(1) | 0.89 | 2.47 | 3.292 | 153 |
| N(2)-H(2E)∙∙∙Br(2) | 0.89 | 2.47 | 3.293 | 153 |
| N(3)-H(3C)∙∙∙Br(2) | 0.97 | 2.92 | 3.876 | 169 |

**Table S9.** Hydrogen bonds data for compound G-[DADPA]PbBr_5_.

| D-H···A | d(D-H) | d(H···A) | d(D···A) | <(DHA) |
| --- | --- | --- | --- | --- |
| N(1)-H(1C)∙∙∙Br(2) | 0.90 | 2.62 | 3.3663 | 140 |
| N(1)-H(1C)∙∙∙Br(4) | 0.90 | 2.90 | 3.3873 | 116 |
| N(1)-H(1D)∙∙∙Br(1) | 0.90 | 2.89 | 3.4491 | 122 |
| N(1)-H(1E)∙∙∙Br(3) | 0.90 | 2.55 | 3.3589 | 149 |
| N(2)-H(2C)∙∙∙Br(1) | 0.90 | 2.78 | 3.3391 | 122 |
| N(2)-H(2C)∙∙∙Br(3) | 0.90 | 2.83 | 3.4254 | 125 |
| N(2)-H(2D)∙∙∙Br(1) | 0.90 | 2.63 | 3.4770 | 157 |
| N(2)-H(2E)∙∙∙Br(2) | 0.90 | 2.62 | 3.4990 | 166 |
| N(3)-H(3C)∙∙∙Br(2) | 0.90 | 2.48 | 3.3028 | 153 |
| N(3)-H(3D)∙∙∙Br(5) | 0.90 | 2.46 | 3.2973 | 155 |
| C(3)-H(3B)∙∙∙Br(5) | 0.97 | 2.93 | 3.8895 | 172 |
| C(6)-H(6B)∙∙∙Br(1) | 0.97 | 2.92 | 3.7601 | 146 |

**Reference**

[1] University of Vienna, Vienna Ab initio Simulation Package (VASP 6.2.0), **2020**, https://www.vasp.at/.

[2] G. Kresse and J. Furthüller, *Phys. Rev. B*, **1996**, *54*, 11169-11186.

[3] G. Kresse and D. Joubert, *Phys. Rev. B*, **1999**, *59*, 1758-1775.

[4] J. P. Perdew, K. Burke and M. Ernzerhof, *Phys. Rev. Lett.*, **1996**, *77*, 3865-3868.

[5] P.E. Blöchl, *Phys. Rev. B*, **1994**, *50*, 17953-17979.

[6] G. Wu, C. Zhou, W. Ming, D. Han, S. Chen, D. Yang, T. Besara, J. Neu, T. Siegrist, M.-H. Du, B. Ma, A. Dong, *ACS Energy Lett* **2018**, *3*, 1443-1449.

[7] J. Zhou, M. Li, L. Ning, R. Zhang, M. S. Molokeev, J. Zhao, S. Yang, K. Han, Z. Xia, *J. Phys. Chem. Lett.* **2019**, *10*, 1337-1341.

[8] M. Li, J. Zhou, G. Zhou, M. S. Molokeev, J. Zhao, V. Morad, M. V. Kovalenko, Z. Xia, *Angew. Chem. Int. Ed.* **2019**, *58*, 18670-18675.

[9] S. Lee, C. Zhou, J. Neu, D. Beery, A. Arcidiacono, M. Chaaban, H. Lin, A. Gaiser, B. Chen, T. E. Albrecht-Schmitt, T. Siegrist, B. Ma, *Chem. Mater.* **2020**, *32*, 374-380.

[10] C. Zhou, H. Lin, J. Neu, Y. Zhou, M. Chaaban, S. Lee, M. Worku, B. Chen, R. Clark, W. Cheng, J. Guan, P. Djurovich, D. Zhang, X. Lü, J. Bullock, C. Pak, M. Shatruk, M.-H. Du, T. Siegrist, B. Ma, *ACS Energy Lett* **2019**, *4*, 1579-1583.

[11] E. R. Dohner, A. Jaffe, L. R. Bradshaw, H. I. Karunadasa, *J. Am. Chem. Soc.* **2014**, *136*, 13154-13157.

[12] T. Ahmed, S. Seth, A. Samanta, *Chem. Mater.* **2018**, *30*, 3633-3637.

[13] M. Imran, V. Caligiuri, M. Wang, L. Goldoni, M. Prato, R. Krahne, L. De Trizio, L. Manna, *J. Am. Chem. Soc.* **2018**, *140*, 2656-2664.

[14] L. Protesescu, S. Yakunin, M. I. Bodnarchuk, F. Bertolotti, N. Masciocchi, A. Guagliardi, M. V. Kovalenko, *J. Am. Chem. Soc.* **2016**, *138*, 14202-14205.

[15] S. Kumar, J. Jagielski, N. Kallikounis, Y.-H. Kim, C. Wolf, F. Jenny, T. Tian, C. J. Hofer, Y.-C. Chiu, W. J. Stark, T.-W. Lee, C.-J. Shih, *Nano Lett.* **2017**, *17*, 5277-5284.

[16] C. Wang, Y. Wang, X. Su, V. G. Hadjiev, S. Dai, Z. Qin, H. A. Calderon Benavides, Y. Ni, Q. Li, J. Jian, M. K. Alam, H. Wang, F. C. Robles Hernandez, Y. Yao, S. Chen, Q. Yu, G. Feng, Z. Wang, J. Bao, *Adv. Mater.* **2019**, *31*, 1902492.

[17] J. Yin, H. Yang, K. Song, A. M. El-Zohry, Y. Han, O. M. Bakr, J.-L. Brédas, O. F. Mohammed, *J. Phys. Chem. Lett.* **2018**, *9*, 5490-5495.

[18] P. Sebastia-Luna, J. Navarro-Alapont, M. Sessolo, F. Palazon, H. J. Bolink, *Chem. Mater.* **2019**, *31*, 10205-10210.

[19] B. Su, M. S. Molokeev, Z. Xia, *J. Mater. Chem. C* **2019**, *7*, 11220-11226.

[20] Q. Kong, X. Meng, S. Ji, Q. Wang, B. Yang, T. Bai, X. Wang, Z. Wang, R. Zhang, D. Zheng, F. Liu, K.-l. Han, *ACS Materials Letters* **2022**, *4*, 1734-1741.

[21] M. Li, J. Zhou, M. S. Molokeev, X. Jiang, Z. Lin, J. Zhao, Z. Xia, *Inorg. Chem.* **2019**, *58*, 13464-13470.

[22] L.-K. Gong, Q.-Q. Hu, F.-Q. Huang, Z.-Z. Zhang, N.-N. Shen, B. Hu, Y. Song, Z.-P. Wang, K.-Z. Du, X.-Y. Huang, *Chem. Commun.* **2019**, *55*, 7303-7306.

[23] Z.-L. He, J.-H. Wei, J.-B. Luo, Z.-Z. Zhang, D.-B. Kuang, *J. Mater. Chem. C* **2023**, *11*, 1251-1257.

[24] L.-J. Xu, C.-Z. Sun, H. Xiao, Y. Wu, Z.-N. Chen, *Adv. Mater.* **2017**, *29*, 1605739.

[25] L.-J. Xu, X. Lin, Q. He, M. Worku, B. Ma, *Nat. Commun.* **2020**, *11*, 4329.

[26] C. Jiang, N. Zhong, C. Luo, H. Lin, Y. Zhang, H. Peng, C.-G. Duan, *Chem. Commun.* **2017**, *53*, 5954-5957.

[27] M.-E. Sun, Y. Li, X.-Y. Dong, S.-Q. Zang, *Chem. Sci.* **2019**, *10*, 3836-3839.

[28] G. Hu, B. Xu, A. Wang, Y. Guo, J. Wu, F. Muhammad, W. Meng, C. Wang, S. Sui, Y. Liu, Y. Li, Y. Zhang, Y. Zhou, Z. Deng, *Adv. Funct. Mater.* **2021**, *31*, 2011191.

[29] V. Morad, I. Cherniukh, L. Pöttschacher, Y. Shynkarenko, S. Yakunin, M. V. Kovalenko, *Chem. Mater.* **2019**, *31*, 10161-10169.
