# Supplementary material for: In Situ Halide Vacancy Tuning of Low‐Dimensional Lead Perovskites to Realize Multiple Adjustable Luminescence Performance
Source: Adv Sci (Weinh). 2025 Mar 17;12(18):2412459. doi: 10.1002/advs.202412459 (PMC12079511; doi:10.1002/advs.202412459)

checkCIF/PLATON report

You have not supplied any structure factors. As a result the full set of tests cannot be run.

THIS REPORT IS FOR GUIDANCE ONLY. IF USED AS PART OF A REVIEW PROCEDURE FOR PUBLICATION, IT SHOULD NOT REPLACE THE EXPERTISE OF AN EXPERIENCED CRYSTALLOGRAPHIC REFEREE.

No syntax errors found.   CIF dictionary   Interpreting this report

Datablock: 1

```
Bond precision:   C-C = 0.0177 A          Wavelength=0.71073

Cell:            a=10.4777(3)    b=11.0286(3)      c=15.3502(5)
                 alpha=90        beta=103.781(1)   gamma=90
Temperature:     273 K

                Calculated                  Reported
Volume          1722.72(9)                  1722.72(9)
Space group     P 21/c                      P 1 21/c 1
Hall group      -P 2ybc                     -P 2ybc
Moiety formula  Br10 Pb2, 2(C6 H20 N3)      Br5 Pb, C6 H20 N3
Sum formula     C12 H40 Br10 N6 Pb2         C6 H20 Br5 N3 Pb
Mr              1481.90                     740.99
Dx,g cm-3       2.857                       2.857
Z               2                           4
Mu (mm-1)       21.371                      21.371
F000            1336.0                      1336.0
F000'           1319.40
h,k,lmax        12,13,18                    12,13,18
Nref            3048                        3046
Tmin,Tmax       0.136,0.118                 0.216,0.746
Tmin'           0.103

Correction method= # Reported T Limits: Tmin=0.216 Tmax=0.746
AbsCorr = NONE

Data completeness= 0.999          Theta(max)= 25.028

R(reflections)= 0.0437( 2644)                 wR2(reflections)=
                                              0.1395( 3046)
S = 1.035                 Npar= 139
```

The following ALERTS were generated. Each ALERT has the format
test-name_ALERT_alert-type_alert-level.
Click on the hyperlinks for more details of the test.

Alert level C

```
PLAT042_ALERT_1_C Calc. and Reported MoietyFormula Strings  Differ     Please Check
              Calc: Br10 Pb2, 2(C6 H20 N3)
              Rep.: Br5 Pb, C6 H20 N3
PLAT234_ALERT_4_C Large Hirshfeld Difference N2       --C3       .       0.17 Ang.
PLAT241_ALERT_2_C High   'MainMol' Ueq as Compared to Neighbors of         C3 Check
PLAT342_ALERT_3_C Low Bond Precision on  C-C Bonds ...............    0.01775 Ang.
PLAT360_ALERT_2_C Short  C(sp3)-C(sp3) Bond  C3       - C6       .       1.38 Ang.
```

Alert level G

```
PLAT003_ALERT_2_G Number of Uiso or Uij Restrained non-H Atoms ...          4 Report
PLAT007_ALERT_5_G Number of Unrefined Donor-H Atoms .............          8 Report
              H1A   H1B   H1C   H2A   H2B   H2C   H3A   H3B
PLAT045_ALERT_1_G Calculated and Reported Z Differ by a Factor ...      0.500 Check
PLAT072_ALERT_2_G SHELXL First  Parameter in WGHT  Unusually Large       0.11 Report
PLAT177_ALERT_4_G The CIF-Embedded .res File Contains DELU Records          2 Report
PLAT192_ALERT_3_G A Non-default DELU Restraint Value for SecondPar     0.0200 Report
PLAT192_ALERT_3_G A Non-default DELU Restraint Value for SecondPar     0.0200 Report
PLAT199_ALERT_1_G Reported _cell_measurement_temperature ..... (K)        273 Check
PLAT200_ALERT_1_G Reported   _diffrn_ambient_temperature ..... (K)        273 Check
PLAT232_ALERT_2_G Hirshfeld Test Diff (M-X)  Pb1      --Br5      .        6.6 s.u.
PLAT232_ALERT_2_G Hirshfeld Test Diff (M-X)  Pb1      --Br1_a    .       24.3 s.u.
PLAT794_ALERT_5_G Tentative Bond Valency for Pb1       (II)      .       2.34 Info
PLAT860_ALERT_3_G Number of Least-Squares Restraints ............          2 Note
```

0 **ALERT level A** = Most likely a serious problem - resolve or explain
0 **ALERT level B** = A potentially serious problem, consider carefully
5 **ALERT level C** = Check. Ensure it is not caused by an omission or oversight
13 **ALERT level G** = General information/check it is not something unexpected

4 ALERT type 1 CIF construction/syntax error, inconsistent or missing data
6 ALERT type 2 Indicator that the structure model may be wrong or deficient
4 ALERT type 3 Indicator that the structure quality may be low
2 ALERT type 4 Improvement, methodology, query or suggestion
2 ALERT type 5 Informative message, check

It is advisable to attempt to resolve as many as possible of the alerts in all categories. Often the minor alerts point to easily fixed oversights, errors and omissions in your CIF or refinement strategy, so attention to these fine details can be worthwhile. In order to resolve some of the more serious problems it may be necessary to carry out additional measurements or structure refinements. However, the purpose of your study may justify the reported deviations and the more serious of these should normally be commented upon in the discussion or experimental section of a paper or in the "special_details" fields of the CIF. checkCIF was carefully designed to identify outliers and unusual parameters, but every test has its limitations and alerts that are not important in a particular case may appear. Conversely, the absence of alerts does not guarantee there are no aspects of the results needing attention. It is up to the individual to critically assess their own results and, if necessary, seek expert advice.

Publication of your CIF in IUCr journals

A basic structural check has been run on your CIF. These basic checks will be run on all CIFs submitted for publication in IUCr journals (*Acta Crystallographica*, *Journal of Applied Crystallography*, *Journal of Synchrotron Radiation*); however, if you intend to submit to *Acta Crystallographica Section C* or *E* or *IUCrData*, you should make sure that full publication checks are run on the final version of your CIF prior to submission.

Publication of your CIF in other journals

Please refer to the *Notes for Authors* of the relevant journal for any special instructions relating to CIF submission.

PLATON version of 06/01/2024; check.def file version of 05/01/2024

Datablock 1 - ellipsoid plot

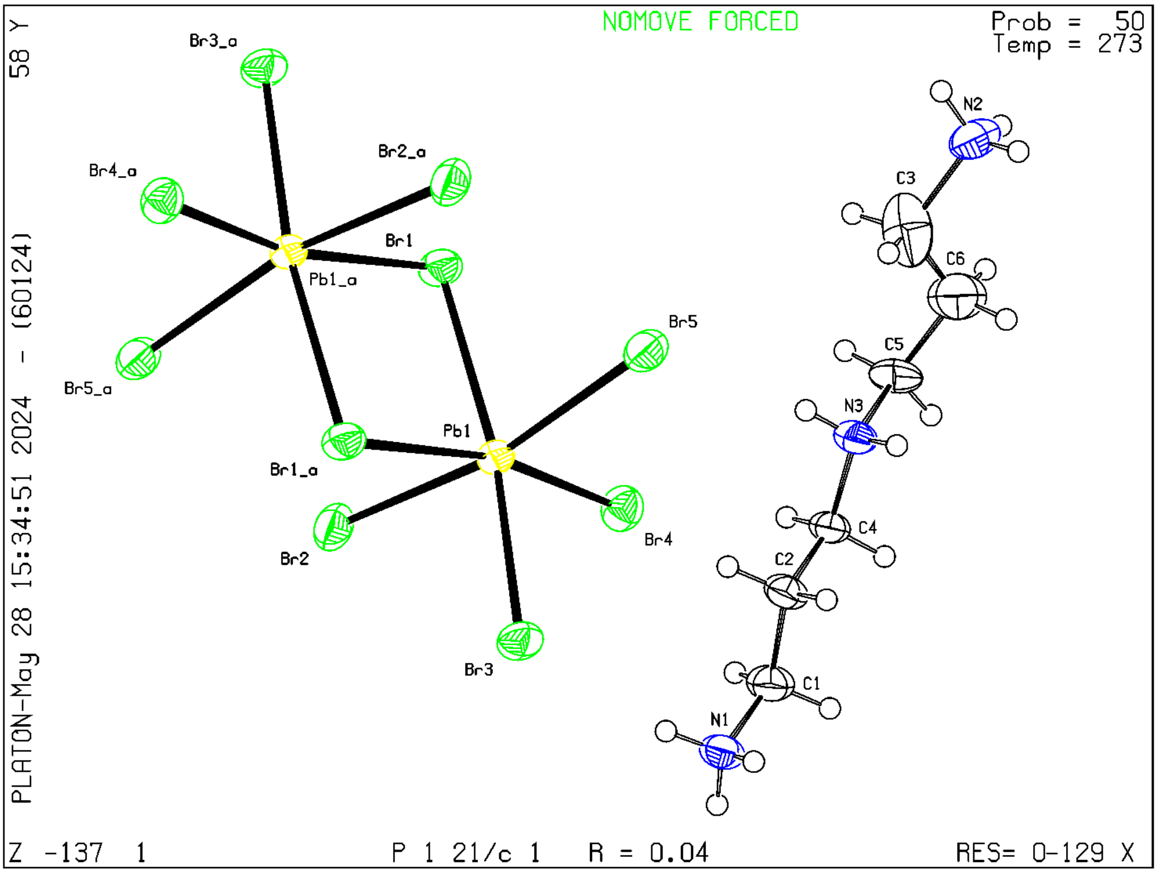

Supplement: Supplementary file 2 — Supporting Information [file ADVS-12-2412459-s002.zip › B-[DADPA]PbBr5-checkcif.pdf]
