# Supplementary material for: In Situ Halide Vacancy Tuning of Low‐Dimensional Lead Perovskites to Realize Multiple Adjustable Luminescence Performance
Source: Adv Sci (Weinh). 2025 Mar 17;12(18):2412459. doi: 10.1002/advs.202412459 (PMC12079511; doi:10.1002/advs.202412459)

checkCIF/PLATON report

Structure factors have been supplied for datablock(s) 1

THIS REPORT IS FOR GUIDANCE ONLY. IF USED AS PART OF A REVIEW PROCEDURE FOR PUBLICATION, IT SHOULD NOT REPLACE THE EXPERTISE OF AN EXPERIENCED CRYSTALLOGRAPHIC REFEREE.

No syntax errors found. CIF dictionary Interpreting this report

Datablock: 1

Bond precision:	C-C = 0.0197 A		Wavelength=0.71073
Cell:	a=10.484(3)	b=11.036(3)	c=15.372(4)
	alpha=90	beta=103.796(5)	gamma=90
Temperature:	296 K		

	Calculated	Reported
Volume	1727.3(8)	1727.3(8)
Space group	P 21/c	P 1 21/c 1
Hall group	-P 2ybc	-P 2ybc
Moiety formula	Br10 Pb2, 2(C6 H20 N3)	4(C6 H20 N3), 4(Br5 Pb)
Sum formula	C12 H40 Br10 N6 Pb2	C24 H80 Br20 N12 Pb4
Mr	1481.90	2963.80
Dx,g cm-3	2.849	2.849
Z	2	1
Mu (mm-1)	21.314	21.315
F000	1336.0	1320.7
F000’	1319.40	
h,k,lmax	13,14,19	13,14,19
Nref	3850	3838
Tmin,Tmax	0.136,0.808	0.399,0.729
Tmin’	0.103	

Correction method= # Reported T Limits: Tmin=0.399 Tmax=0.729
AbsCorr = MULTI-SCAN

Data completeness= 0.997 Theta(max)= 27.180

R(reflections)= 0.0563(2436) wR2(reflections)= 0.1421(3838)

S = 0.974 Npar= 131

The following ALERTS were generated. Each ALERT has the format
test-name_ALERT_alert-type_alert-level.
Click on the hyperlinks for more details of the test.

Alert level C

```
PLAT041_ALERT_1_C Calc. and Reported SumFormula    Strings  Differ     Please Check
              Calc: C12 H40 Br10 N6 Pb2
              Rep.: C24 H80 Br20 N12 Pb4
PLAT042_ALERT_1_C Calc. and Reported MoietyFormula Strings  Differ     Please Check
              Calc: Br10 Pb2, 2(C6 H20 N3)
              Rep.: 4(C6 H20 N3), 4(Br5 Pb)
PLAT068_ALERT_1_C Reported F000 Differs from Calcd (or Missing)...     Please Check
PLAT202_ALERT_3_C Isotropic non-H Atoms in Anion/Solvent .........          1 Check
              C2
PLAT241_ALERT_2_C High   'MainMol' Ueq as Compared to Neighbors of         C2 Check
PLAT241_ALERT_2_C High   'MainMol' Ueq as Compared to Neighbors of         C7 Check
PLAT342_ALERT_3_C Low Bond Precision on  C-C Bonds ...............    0.01975 Ang.
PLAT905_ALERT_3_C Negative K value in the Analysis of Variance ...     -0.050 Report
PLAT911_ALERT_3_C Missing FCF Refl Between Thmin & STh/L=    0.600          2 Report
                2  0 16,   3  0 16,
PLAT925_ALERT_1_C The Reported and Calculated Rho(max) Differ by .       1.21 eA-3
PLAT971_ALERT_2_C Check Calcd Resid. Dens.  1.08Ang From C1              1.58 eA-3
PLAT972_ALERT_2_C Check Calcd Resid. Dens.  0.73Ang From Pb1            -2.32 eA-3
PLAT972_ALERT_2_C Check Calcd Resid. Dens.  0.77Ang From Pb1            -2.25 eA-3
PLAT972_ALERT_2_C Check Calcd Resid. Dens.  0.76Ang From Pb1            -2.15 eA-3
PLAT972_ALERT_2_C Check Calcd Resid. Dens.  0.77Ang From Pb1            -2.13 eA-3
PLAT972_ALERT_2_C Check Calcd Resid. Dens.  0.86Ang From Pb1            -1.77 eA-3
PLAT972_ALERT_2_C Check Calcd Resid. Dens.  0.87Ang From Pb1            -1.67 eA-3
PLAT972_ALERT_2_C Check Calcd Resid. Dens.  0.82Ang From Br5            -1.62 eA-3
PLAT977_ALERT_2_C Check Negative Difference Density on H1B       .      -0.72 eA-3
PLAT977_ALERT_2_C Check Negative Difference Density on H1E       .      -0.40 eA-3
PLAT977_ALERT_2_C Check Negative Difference Density on H2B       .      -0.46 eA-3
PLAT977_ALERT_2_C Check Negative Difference Density on H2D       .      -0.70 eA-3
PLAT977_ALERT_2_C Check Negative Difference Density on H3B       .      -0.32 eA-3
PLAT977_ALERT_2_C Check Negative Difference Density on H8A       .      -0.65 eA-3
```

Alert level G

```
PLAT002_ALERT_2_G Number of Distance or Angle Restraints on AtSite          4 Note
PLAT003_ALERT_2_G Number of Uiso or U(i,j)  Restrained non-H-Atoms          2 Report
PLAT045_ALERT_1_G Calculated and Reported Z Differ by a Factor ...          2 Check
PLAT073_ALERT_1_G H-atoms ref., but hydrogen treatment Reported as     constr Check
PLAT172_ALERT_4_G The CIF-Embedded .res File Contains DFIX Records          3 Report
PLAT177_ALERT_4_G The CIF-Embedded .res File Contains DELU Records          1 Report
PLAT192_ALERT_3_G A Non-default DELU Restraint Value for SecondPar     0.0200 Report
PLAT232_ALERT_2_G Hirshfeld Test Diff (M-X)  Pb1       --Br1     .        5.2 s.u.
PLAT232_ALERT_2_G Hirshfeld Test Diff (M-X)  Pb1       --Br2_a   .       18.7 s.u.
PLAT768_ALERT_4_G Embedded RES Explicitly Supplied Scattering Data     Please Note
PLAT769_ALERT_4_G CIF Embedded Explicitly Supplied Scattering Data     Please Note
PLAT794_ALERT_5_G Tentative Bond Valency for Pb1        (II)       .       2.32 Info
PLAT860_ALERT_3_G Number of Least-Squares Restraints .............          4 Note
PLAT912_ALERT_4_G Missing # of FCF Reflections Above STh/L=  0.600         12 Note
PLAT960_ALERT_3_G Number of Intensities with I < - 2*Sigma(I) ....          9 Check
PLAT969_ALERT_5_G The 'Henn et al.' R-Factor-gap value ..........       1.326 Note
              Predicted wR2: Based on SigI**2 10.72 or SHELX Weight 14.58
```

```
PLAT978_ALERT_2_G Number C-C Bonds with Positive Residual Density.          0 Info
```

```
   0 ALERT level A = Most likely a serious problem - resolve or explain
   0 ALERT level B = A potentially serious problem, consider carefully
  24 ALERT level C = Check. Ensure it is not caused by an omission or oversight
  17 ALERT level G = General information/check it is not something unexpected

   6 ALERT type 1 CIF construction/syntax error, inconsistent or missing data
  21 ALERT type 2 Indicator that the structure model may be wrong or deficient
   7 ALERT type 3 Indicator that the structure quality may be low
   5 ALERT type 4 Improvement, methodology, query or suggestion
   2 ALERT type 5 Informative message, check
```

It is advisable to attempt to resolve as many as possible of the alerts in all categories. Often the minor alerts point to easily fixed oversights, errors and omissions in your CIF or refinement strategy, so attention to these fine details can be worthwhile. In order to resolve some of the more serious problems it may be necessary to carry out additional measurements or structure refinements. However, the purpose of your study may justify the reported deviations and the more serious of these should normally be commented upon in the discussion or experimental section of a paper or in the "special_details" fields of the CIF. checkCIF was carefully designed to identify outliers and unusual parameters, but every test has its limitations and alerts that are not important in a particular case may appear. Conversely, the absence of alerts does not guarantee there are no aspects of the results needing attention. It is up to the individual to critically assess their own results and, if necessary, seek expert advice.

Publication of your CIF in IUCr journals

A basic structural check has been run on your CIF. These basic checks will be run on all CIFs submitted for publication in IUCr journals (*Acta Crystallographica*, *Journal of Applied Crystallography*, *Journal of Synchrotron Radiation*); however, if you intend to submit to *Acta Crystallographica Section C* or *E* or *IUCrData*, you should make sure that full publication checks are run on the final version of your CIF prior to submission.

Publication of your CIF in other journals

Please refer to the *Notes for Authors* of the relevant journal for any special instructions relating to CIF submission.

PLATON version of 11/11/2024; check.def file version of 11/11/2024

Datablock 1 - ellipsoid plot

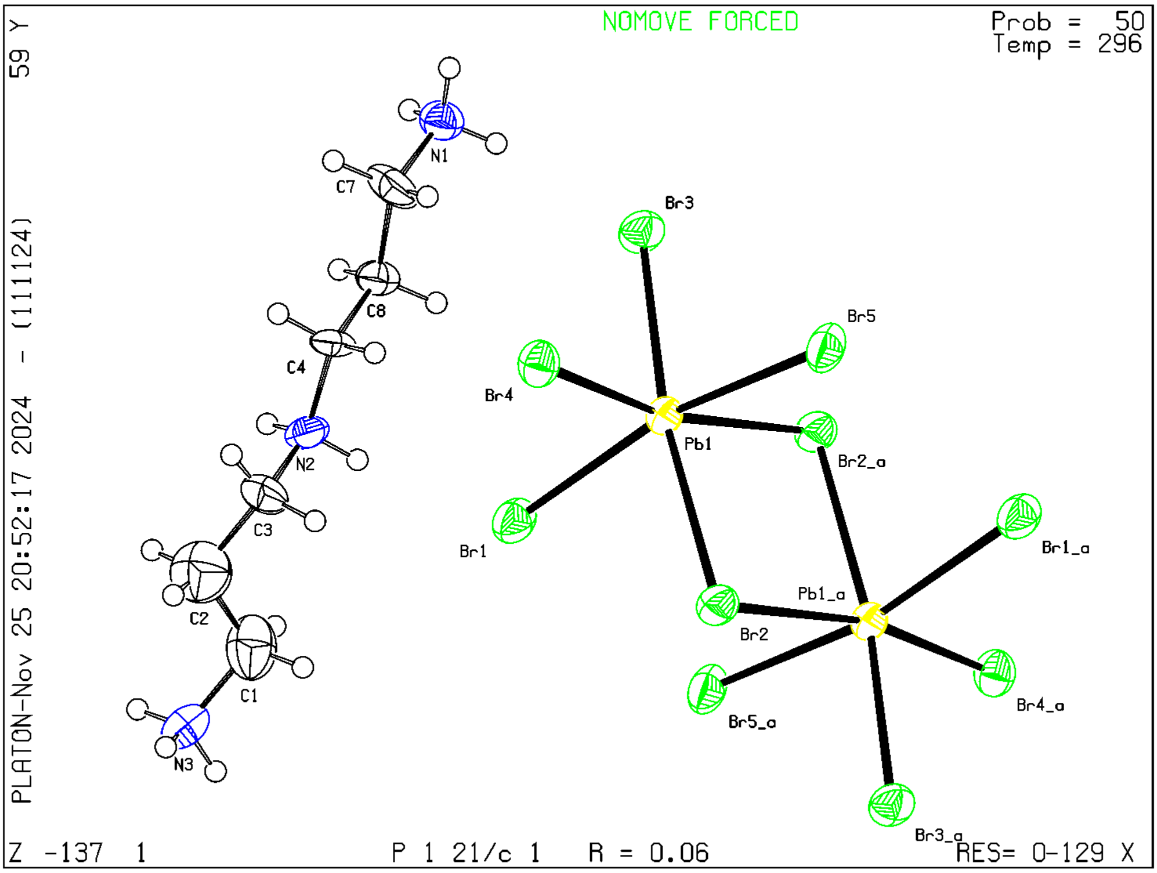

Supplement: Supplementary file 2 — Supporting Information [file ADVS-12-2412459-s002.zip › G-[DADPA]PbBr5-checkcif.pdf]
