# Supplementary material for: In Situ Halide Vacancy Tuning of Low‐Dimensional Lead Perovskites to Realize Multiple Adjustable Luminescence Performance
Source: Adv Sci (Weinh). 2025 Mar 17;12(18):2412459. doi: 10.1002/advs.202412459 (PMC12079511; doi:10.1002/advs.202412459)

checkCIF/PLATON report

Structure factors have been supplied for datablock(s) 1

THIS REPORT IS FOR GUIDANCE ONLY. IF USED AS PART OF A REVIEW PROCEDURE FOR PUBLICATION, IT SHOULD NOT REPLACE THE EXPERTISE OF AN EXPERIENCED CRYSTALLOGRAPHIC REFEREE.

No syntax errors found. CIF dictionary Interpreting this report

Datablock: 1

Bond precision: C-C = 0.0162 A Wavelength=0.71073

Cell:	a=10.4829(19)	b=11.038(2)	c=15.373(3)
	alpha=90	beta=103.763(2)	gamma=90
Temperature:	296 K		

	Calculated	Reported
Volume	1727.7(6)	1727.7(6)
Space group	P 21/c	P 1 21/c 1
Hall group	-P 2ybc	-P 2ybc
Moiety formula	Br10 Pb2, 2(C6 H20 N3)	4(C6 H20 N3), 4(Br5 Pb)
Sum formula	C12 H40 Br10 N6 Pb2	C24 H64 Br20 N12 Pb4
Mr	1481.90	2947.67
Dx,g cm-3	2.849	2.833
Z	2	1
Mu (mm-1)	21.309	21.308
F000	1336.0	1320.0
F000'	1319.40	
h,k,lmax	13,14,20	13,14,19
Nref	3987	3975
Tmin,Tmax	0.141,0.808	0.399,0.729
Tmin'	0.109	

Correction method= # Reported T Limits: Tmin=0.399 Tmax=0.729
AbsCorr = MULTI-SCAN

Data completeness= 0.997 Theta(max)= 27.548

R(reflections)= 0.0433(2627) wR2(reflections)= 0.0916(3975)

S = 0.997 Npar= 133

The following ALERTS were generated. Each ALERT has the format
test-name_ALERT_alert-type_alert-level.
Click on the hyperlinks for more details of the test.

Alert level C

```
PLAT041_ALERT_1_C Calc. and Reported SumFormula    Strings  Differ     Please Check
              Calc: C12 H40 Br10 N6 Pb2
              Rep.: C24 H64 Br20 N12 Pb4
PLAT042_ALERT_1_C Calc. and Reported MoietyFormula Strings  Differ     Please Check
              Calc: Br10 Pb2, 2(C6 H20 N3)
              Rep.: 4(C6 H20 N3), 4(Br5 Pb)
PLAT043_ALERT_1_C Calculated and Reported Mol. Weight Differ by ..       8.07 Check
PLAT202_ALERT_3_C Isotropic non-H Atoms in Anion/Solvent .........          1 Check
              C1
PLAT230_ALERT_2_C Hirshfeld Test Diff for    N2       --C5       .        6.7 s.u.
PLAT241_ALERT_2_C High   'MainMol' Ueq as Compared to Neighbors of         C1 Check
PLAT241_ALERT_2_C High   'MainMol' Ueq as Compared to Neighbors of         C5 Check
PLAT342_ALERT_3_C Low Bond Precision on  C-C Bonds ...............    0.01625 Ang.
PLAT360_ALERT_2_C Short  C(sp3)-C(sp3) Bond  C1        - C5       .      1.35 Ang.
PLAT906_ALERT_3_C Large K Value in the Analysis of Variance ......      2.097 Check
PLAT911_ALERT_3_C Missing FCF Refl Between Thmin & STh/L=    0.600          2 Report
               -2  0 18,  -1  0 18,
PLAT971_ALERT_2_C Check Calcd Resid. Dens.  1.20Ang From C5                1.79 eA-3
PLAT975_ALERT_2_C Check Calcd Resid. Dens.  0.96Ang From N1       .        0.67 eA-3
PLAT977_ALERT_2_C Check Negative Difference Density on H1A        .       -0.50 eA-3
PLAT977_ALERT_2_C Check Negative Difference Density on H2A        .       -0.31 eA-3
PLAT977_ALERT_2_C Check Negative Difference Density on H2C        .       -0.35 eA-3
```

Alert level G

```
FORMU01_ALERT_1_G  There is a discrepancy between the atom counts in the
            _chemical_formula_sum and _chemical_formula_moiety. This is
            usually due to the moiety formula being in the wrong format.
            Atom count from _chemical_formula_sum:   C24 H64 Br20 N12 Pb4
            Atom count from _chemical_formula_moiety:C24 H80 Br20 N12 Pb4
FORMU01_ALERT_2_G  There is a discrepancy between the atom counts in the
            _chemical_formula_sum and the formula from the _atom_site* data.
            Atom count from _chemical_formula_sum:C24 H64 Br20 N12 Pb4
            Atom count from the _atom_site data:  C24 H80 Br20 N12 Pb4
CELLZ01_ALERT_1_G Difference between formula and atom_site contents detected.
CELLZ01_ALERT_1_G ALERT: Large difference may be due to a
            symmetry error - see SYMMG tests
           From the CIF: _cell_formula_units_Z    1
           From the CIF: _chemical_formula_sum  C24 H64 Br20 N12 Pb4
           TEST: Compare cell contents of formula and atom_site data

           atom    Z*formula  cif sites diff
           C         24.00     24.00    0.00
           H         64.00     80.00  -16.00
           Br        20.00     20.00    0.00
           N         12.00     12.00    0.00
           Pb         4.00      4.00    0.00
PLAT007_ALERT_5_G Number of Unrefined Donor-H Atoms .............          8 Report
              H1A   H1B   H1C   H2A   H2B   H2C   H3A   H3B
PLAT045_ALERT_1_G Calculated and Reported Z Differ by a Factor ...          2 Check
```

```
PLAT068_ALERT_1_G Reported F000 Differs from Calcd (or Missing)...     Please Check
PLAT232_ALERT_2_G Hirshfeld Test Diff (M-X)  Pb1      --Br1      .        7.0 s.u.
PLAT232_ALERT_2_G Hirshfeld Test Diff (M-X)  Pb1      --Br2      .        5.0 s.u.
PLAT232_ALERT_2_G Hirshfeld Test Diff (M-X)  Pb1      --Br1_a    .       28.0 s.u.
PLAT768_ALERT_4_G Embedded RES Explicitly Supplied Scattering Data     Please Note
PLAT774_ALERT_1_G Check   X-Y Bond in CIF: Pb1      --Pb1       ..       4.42 Ang.
PLAT794_ALERT_5_G Tentative Bond Valency for Pb1       (II)      .       2.31 Info
PLAT883_ALERT_1_G Absent Datum for _atom_sites_solution_primary ..     Please Do !
PLAT912_ALERT_4_G Missing # of FCF Reflections Above STh/L=  0.600          9 Note
PLAT969_ALERT_5_G The 'Henn et al.' R-Factor-gap value ..........      1.410 Note
              Predicted wR2: Based on SigI**2  6.50 or SHELX Weight  9.18
PLAT978_ALERT_2_G Number C-C Bonds with Positive Residual Density.          0 Info
```

```
   0 ALERT level A = Most likely a serious problem - resolve or explain
   0 ALERT level B = A potentially serious problem, consider carefully
  16 ALERT level C = Check. Ensure it is not caused by an omission or oversight
  17 ALERT level G = General information/check it is not something unexpected

  10 ALERT type 1 CIF construction/syntax error, inconsistent or missing data
  14 ALERT type 2 Indicator that the structure model may be wrong or deficient
   4 ALERT type 3 Indicator that the structure quality may be low
   2 ALERT type 4 Improvement, methodology, query or suggestion
   3 ALERT type 5 Informative message, check
```

It is advisable to attempt to resolve as many as possible of the alerts in all categories. Often the minor alerts point to easily fixed oversights, errors and omissions in your CIF or refinement strategy, so attention to these fine details can be worthwhile. In order to resolve some of the more serious problems it may be necessary to carry out additional measurements or structure refinements. However, the purpose of your study may justify the reported deviations and the more serious of these should normally be commented upon in the discussion or experimental section of a paper or in the "special_details" fields of the CIF. checkCIF was carefully designed to identify outliers and unusual parameters, but every test has its limitations and alerts that are not important in a particular case may appear. Conversely, the absence of alerts does not guarantee there are no aspects of the results needing attention. It is up to the individual to critically assess their own results and, if necessary, seek expert advice.

Publication of your CIF in IUCr journals

A basic structural check has been run on your CIF. These basic checks will be run on all CIFs submitted for publication in IUCr journals (*Acta Crystallographica*, *Journal of Applied Crystallography*, *Journal of Synchrotron Radiation*); however, if you intend to submit to *Acta Crystallographica Section C* or *E* or *IUCrData*, you should make sure that full publication checks are run on the final version of your CIF prior to submission.

Publication of your CIF in other journals

Please refer to the *Notes for Authors* of the relevant journal for any special instructions relating to CIF submission.

PLATON version of 11/11/2024; check.def file version of 11/11/2024

Datablock 1 - ellipsoid plot

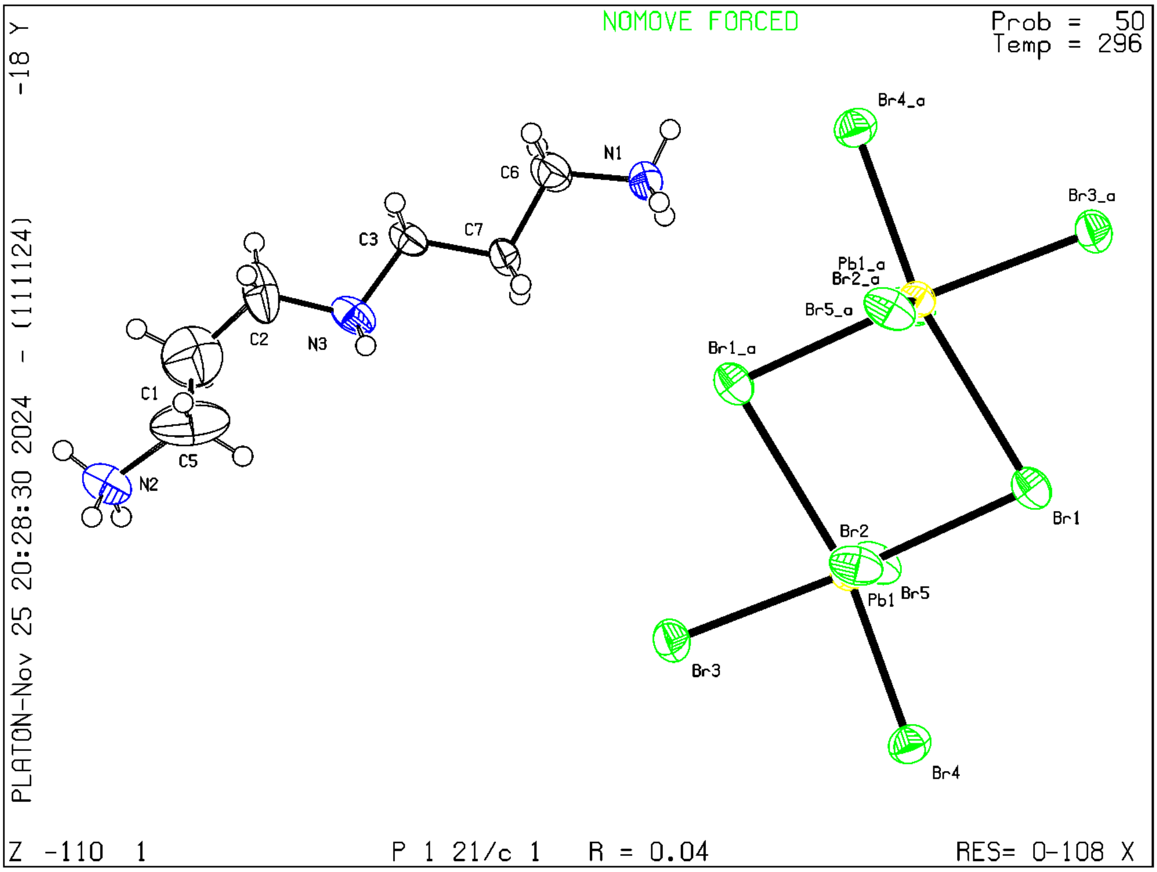

Supplement: Supplementary file 2 — Supporting Information [file ADVS-12-2412459-s002.zip › W-[DADPA]PbBr5-checkcif.pdf]
